# Comparative molecular analyses of select pH- and osmoregulatory genes in three freshwater crayfish *Cherax quadricarinatus*, *C. destructor* and *C. cainii*

**DOI:** 10.7717/peerj.3623

**Published:** 2017-08-24

**Authors:** Muhammad Y. Ali, Ana Pavasovic, Lalith K. Dammannagoda, Peter B. Mather, Peter J. Prentis

**Affiliations:** 1 School of Earth, Environmental and Biological Sciences, Queensland University of Technology, Brisbane, Queensland, Australia; 2 School of Biomedical Sciences, Queensland University of Technology, Brisbane, Queensland, Australia

**Keywords:** Redclaw, Yabby, Marron, Osmoregulatory genes, V-type H^+^-ATPase, Carbonic anhydrase, Na^+^/K^+^-ATPase

## Abstract

Systemic acid-base balance and osmotic/ionic regulation in decapod crustaceans are in part maintained by a set of transport-related enzymes such as carbonic anhydrase (CA), Na^+^/K^+^-ATPase (NKA), H^+^-ATPase (HAT), Na^+^/K^+^/2Cl^−^ cotransporter (NKCC), Na^+^/Cl^−^/HCO${}_{3}^{-}$ cotransporter (NBC), Na^+^/H^+^ exchanger (NHE), Arginine kinase (AK), Sarcoplasmic Ca^+2^-ATPase (SERCA) and Calreticulin (CRT). We carried out a comparative molecular analysis of these genes in three commercially important yet eco-physiologically distinct freshwater crayfish*, Cherax quadricarinatus, C. destructor* and * C. cainii*, with the aim to identify mutations in these genes and determine if observed patterns of mutations were consistent with the action of natural selection. We also conducted a tissue-specific expression analysis of these genes across seven different organs, including gills, hepatopancreas, heart, kidney, liver, nerve and testes using NGS transcriptome data. The molecular analysis of the candidate genes revealed a high level of sequence conservation across the three *Cherax sp.* Hyphy analysis revealed that all candidate genes showed patterns of molecular variation consistent with neutral evolution. The tissue-specific expression analysis showed that 46% of candidate genes were expressed in all tissue types examined, while approximately 10% of candidate genes were only expressed in a single tissue type. The largest number of genes was observed in nerve (84%) and gills (78%) and the lowest in testes (66%). The tissue-specific expression analysis also revealed that most of the master genes regulating pH and osmoregulation (CA, NKA, HAT, NKCC, NBC, NHE) were expressed in all tissue types indicating an important physiological role for these genes outside of osmoregulation in other tissue types. The high level of sequence conservation observed in the candidate genes may be explained by the important role of these genes as well as potentially having a number of other basic physiological functions in different tissue types.

## Introduction

In decapod crustaceans, systematic acid–base balance and ion-regulation are processes that are largely controlled by a set of transport-related enzymes. These enzymes include carbonic anhydrase (CA), Na^+^/K^+^-ATPase (NKA), Vacuolar-type H^+^-ATPase (HAT), Na^+^/K^+^/2Cl^−^ cotransporter (NKCC), Na^+^/HCO${}_{3}^{-}$ cotransporter (NBC), Arginine kinase (AK), Calreticulin (CRT), Sarco/endoplasmic reticulum Ca^2+^ATPase (SERCA), Na^+^/H^+^ exchanger (NHE) and Na^+^/Ca^+2^ exchanger (NCX) (Pan, Zhang & Liu, 2007; Serrano, Halanych & Henry, 2007; Freire, Onken & McNamara, 2008; Henry et al., 2012; McNamara & Faria, 2012; Romano & Zeng, 2012; Havird, Henry & Wilson, 2013). The expression patterns and activities of these genes/enzymes have been reported in a number of decapod crustaceans (for example; CA (Serrano, Halanych & Henry, 2007; Pongsomboon et al., 2009; Ali et al., 2015b; Liu et al., 2015); NKA (Chaudhari et al., 2015; Han et al., 2015; Leone et al., in press); HAT (Pan, Zhang & Liu, 2007; Wang et al., 2012; Havird, Santos & Henry, 2014; Ali et al., 2015b); NKCC (Towle et al., 1997; Havird, Henry & Wilson, 2013; Havird, Santos & Henry, 2014); NHE (Towle & Weihrauch, 2001; Weihrauch et al., 2004; Ren et al., 2015); NCX (Flik et al., 1994; Lucu & Flik, 1999; Flik & Haond, 2000) AK (Serrano, Halanych & Henry, 2007; Havird, Santos & Henry, 2014; Ali et al., 2015b); CRT (Luana et al., 2007; Visudtiphole et al., 2010; Watthanasurorot et al., 2013; Duan et al., 2014). Despite this, we know little about the molecular evolution of these key gene classes or their patterns of tissue specific expression in decapod crustaceans.

*Cherax quadricarinatus* (Redclaw), *Cherax destructor* (Yabby) and *Cherax cainii* (Marron) are commercially important freshwater crayfish, endemic to Australia. These species occur in a diverse range of aquatic environments and can tolerate wide fluctuations in a range of water parameters such as pH, salinity, dissolved oxygen and temperature (Bryant & Papas, 2007; McCormack, 2014). This indicates that these crayfish species have a great capacity to cope with highly variable aquatic environments. Consequently, because of the physiological robustness of these animals and their economic significance, it has led to their use in physiological genomic research in crustaceans.

In previous studies we have identified a number of the above mentioned ion-transport related genes in *C. quadricarinatus*, *C. destructor* and *C. cainii* (Ali et al., 2015a; Ali et al., 2015b). In the present study, we undertook an in-depth molecular analysis of candidate systemic acid–base and ion-transport related genes across these three freshwater crayfish species. In addition, we have performed comparative and evolutionary genomic analysis of these genes across other related arthropod species. The main focus of this study was to identify synonymous and non-synonymous mutations in these genes and to determine if the observed patterns of mutations were consistent with the action of natural selection or neutral evolution. This study also investigated patterns of tissue-specific expression of candidate pH and ion/osmoregulatory genes in the gills, hepatopancreas, heart, kidney, liver, nervous system and testes using publically available and newly generated transcriptome metadata.

## Material and Methods

We sequenced gills and hepatopancreas in your lab and other data came from other sources.

### Sample collection and preparation

Live redclaw crayfish (*C. quadricarinatus*) were obtained from Theebine, in Queensland, Australia; yabby (*C. destructor*) were sourced from New South Wales, Australia; and Marron (*C. cainii*) from Mudgee, Western Australia, Australia. Culture conditions for *C. quadricarinatus* has already been described in (Ali et al., 2015b). *Cherax destructor* and *C. cainii* were housed at QUT aquaculture facility under standard culture conditions. Animals were acclimated to lab conditions for three weeks (21 °C, pH 7 and conductivity 1700 µS/cm). Before tissue collection, three *C. cainii* (body length 11 ± 0.9 cm and wet body weight 57 ± 5g) and three *C. destructor* (9 ± 0.7 cm and 45 ± 3g) were exposed to pH 6, 7 and 8 (for 6 h); a natural pH tolerance range of *Cherax* species (Macaranas et al., 1995; Bryant & Papas, 2007). The animals were exposed to different pH for 6 h because our previous study shows that different isoforms of some the genes of our interest (for example, cytoplasmic isoform of Carbonic anhydrase CAc and membrane-associated isoform CAg) expressed differently after approximately 6 h (Ali et al., 2015b).

### RNA extraction, cDNA synthesis and sequencing

Gills were excised from *C. destructor* and *C. cainii* samples, immediately following euthanisation in ice for 5–10 min. Tissues were extracted after six hours at each treatment, following euthanisation in ice-water. Tissue samples across the three treatments were pooled for each species separately and crushed in liquid nitrogen before total RNA was extracted using an existing protocol (Prentis & Pavasovic, 2014). Genomic DNA was digested with Turbo DNA-free kit (Life Technologies, Carlsbad, CA, USA) and RNA quality and concentration was determined using both Bioanalyzer 2100 RNA nanochip (Agilent Technologies, Santa Clara, CA, USA) and NanoDrop 2000 (Thermo Scientific, Waltham, MA, USA). RNASeq library preparation and paired-end sequencing were undertaken on an Illumina NextSeq 500 (150 bp pair-end chemistry) according to the manufacturer’s protocol for stranded library preparation.

Six individual hepatopancreas samples from *C. quadricarinatus* were dissected and homogenised in liquid N_2_ before total RNA was extracted using the protocol of Prentis & Pavasovic (2014). Genomic DNA was digested with Turbo DNA-free kit (REF-AM1907, Ambion RNA; Life Technologies, Carlsbad, CA, USA) and RNA quality and concentration were checked using the Bioanalyzer 2100 (Agilent Technologies, Santa Clara, CA, USA). RNASeq library preparation and sequencing were undertaken according to the Ion Proton 200 bp library preparation and sequencing protocol (Thermo Fisher, see Amin et al., 2014). Raw Illumina sequence read data for heart, kidney, liver, nerve and testes were downloaded from NCBI Sequence Read Archive database as FastQ files (Accession SRA: ERR391748 –ERR391752 Tan et al., 2016).

### Assembly, functional annotation and gene identification

Reads which did not meet our quality criteria (Q>30, N bases <1%) were removed before assembly using Trimmomatic (Version 0.32; Bolger, Lohse & Usadel, 2014). These filtered reads were assembled with the Trinity short read *de novo* assembler using the stranded option for Illumina data (Version 2014-04-13p1; Haas et al., 2013) and the unstranded option for Ion Torrent data. Contigs with >95% sequence similarity were clustered into gene families using CD-HIT (Version 4.6.1; Huang et al., 2010). Coding sequences representing open reading frames (ORFs) from the transcripts were determined using Transdecoder (Version r20140704; Haas et al., 2013).

Assembled data were used as BLASTx queries against the NCBI NR protein database using BLAST+ (Version 2.2.29; Camacho et al., 2009) with a stringency of 10 × *e*^−5^. Gene ontology (GO) terms were assigned to annotated transcripts using Blast2Go Pro (Version 3.0; Conesa et al., 2005). Candidate genes were identified based on literature searches, PFAM domains, and GO terms (Henry et al., 2012; Eissa & Wang, 2014; Ali et al., 2015b).

### Molecular analyses

Comparative molecular analyses were carried out on the candidate genes previously reported to have important role in pH balance and/or ion-/osmo-regulation. The translated amino acids sequences from translated ORFs were used as BLASTp queries against the NCBI NR database. The top BLAST hits were downloaded for each gene and sequences were aligned in BioEdit (version 7.2.5; Hall, 1999) using a ClustalW alignment (Larkin et al., 2007). Neighbour Joining trees based on amino acid data were generated in Geneious (version 8.0.4) using the Jukes Cantor method with 10,000 bootstraps (Kearse et al., 2012). Important functional residues and PFAM domains in the predicted amino acid sequences for each candidate gene were determined using SMART (Schultz et al., 1998), PROSITE (Sigrist et al., 2013) and NCBI’s Conserved Domain Database (Marchler-Bauer et al., 2015). Putative signal peptides, functional motifs and potential cleavage sites of signal peptides were predicted using PrediSi (PrediSI, 2014) and SignalP 4.0 (Petersen et al., 2011). N-linked glycosylation sites were predicted with N-GlycoSite (Zhang et al., 2004) using the NXS/T model (where N, Aspargine; S, Serine; T, Theronine and X, any amino acid).

Molecular evolutionary analyses for each of the gene sequences were generated using MEGA (version 6; Tamura et al., 2013). Estimates of natural selection for each codon (codon-by-codon) were determined using HyPHy analysis (hypothesis testing using phylogenies) according to (Sergei & Muse, 2005). In this analysis, estimates of the numbers of synonymous (s) and nonsynonymous (n) substitutions for each codon, and their respective potential numbers of synonymous (S) and nonsyonymous (N) sites were also determined. These estimates were calculated using joint Maximum Likelihood reconstructions of ancestral states under a Muse-Gaut model (Muse & Gaut, 1994) of codon substitution and a General Time Reversible model (Nei & Kumar, 2000). The differences between the nonsynonymous substitutions rate (per site) (dN = n/N) and synonymous substitutions rate (dS = s/S) were used for detecting the codons that had undergone positive selection. The null hypothesis of neutral evolution was rejected at *p* < 0.05 (Suzuki & Gojobori, 1999; Kosakovsky & Frost, 2005). All positions in the aligned sequences containing gaps and missing data were eliminated from the final analysis.

Estimates of evolutionary divergence between the sequences were conducted in MEGA6 using the Poisson correction model (Zuckerkandl & Pauling, 1965). The equality ofevolutionary rate between two sequences from two different species was conducted according to Tajima’s relative rate test using amino acid substitutions model (Tajima, 1993).

### Tissue-specific expression

Transcriptomes were assembled separately for seven different *C. quadricarinatus* organs including gills, hepatopancreas, heart, kidney, liver, nerve and testes. This analysis examined the presence or absence of 80 important genes that are directly or indirectly involved in either acid–base balance or osmotic/ionic regulation across the seven different tissues. Two data sets (from gills and hepatopancreas) were generated in our lab and the remaining five were sourced from publicly available NGS-sequenced data archived in NCBI SRA data bank (Tan et al., 2016).

## Results

### Transcriptome assembly, annotation and gene identification

#### Transcriptomes of gills from Yabby and Marron

RNA libraries yielded more than 83 million (83,984,583) and 100 million (100,712,892) high quality (Q ≥ 30) 150 bp paired-end reads for *C. cainii* and *C. destructor,* respectively. Assemblies resulted in 147,101 contigs (*C. cainii*) and 136,622 contigs (*C. destructor*) ≥ 200 bp. Contigs with >70% protein sequence similarity were clustered into 18,325 and 18,113 gene families in *C. cainii* and *C. destructor* (Table 1). Average contig length (747 and 740) and the N50 statistic (1,380 and 1,326), which is a weighted median statistic where half of the entire assembly length is contained in contigs equal to or larger than this value, were similar in both assemblies. Average contig lengths from our assemblies were longer than that from other assemblies in crustaceans (e.g., 323 bp in *Macrobrachium rosenbergii*, (Mohd Shamsudin et al., 2013) and 492 bp in *Euphausia superba* (Clark et al., 2011)).

**Table 1:** Summary statistics for assembled contigs from different organs of *C. quadricarinatus*, *C. destructor* and *C. cainii*. Summary statistics for assembled contigs greater than 200 bp generated from different organs of *Cherax quadricarinatus* (Redclaw), *C. destructor* (Yabby) and *C. cainii* (Marron) using Trinity *de novo* assembler and cd-HIT clustering tool.

Summary statistics	Redclaw							Yabby	Marron
	Gills	Hepato- pancreas	Heart	Kidney	Liver	Nerve	Testes	Gills	Gills
Total reads (million)	72.3	65	47	63	51	64	62	100	83.9
Number of total contigs	87,290	67,401	38,938	66,308	47,166	66,564	63,924	136,622	147,101
N50	725	398	720	1,033	705	1,190	995	1,326	1,380
Mean contig length	563	386	554	663	548	716	653	740	747
Length of the longest contig	15,028	9,532	16,343	15,021	17,690	20,121	16,889	17,725	22,324
Number of contigs longer than 500 bp	27,973	12,055	10,129	22,622	13,665	23,290	19,642	49,678	52,459
Number of contigs longer than 1,500 bp	5,274	0	2,048	6,264	2,867	6,670	5,050	16,178	17,486
Number of clusters	24,123	21,732	23,127	19,816	22,963	22,837	22,989	18,113	18,325
Blast Success rate (%)	25.3	35	31.2	25.9	27.7	27.1	27.4	17	18

More than 23,000 contigs for each species received significant BLASTx hits against the NR database with a stringency of e-^5^. The percentage BLAST success attained for the two transcriptomes (17–18%) were lower compared to other freshwater crayfish; *Procambarus clarkii* (36%; Shen et al., 2014) and *C. quadricarinatus* (37%, Ali et al., 2015b).

#### Transcriptomes of different organs from Redclaw (*C. quadricarinatus*)

The seven transcriptome libraries, when combined, constituted a data set of 608 million high quality (Q ≥ 30) reads. RNA libraries for gills yielded more than 72 million (72,382,710), 83 million (83,984,583) and 100 million (100,712,892) high quality reads for *C. quadricarinatus*, *C. cainii* and *C. destructor,* respectively. The hepatopancreas transcriptome from Redclaw yielded ≈65 million reads and 67,401 transcripts (Table 1). The detailed statistics of raw reads, assembled contigs and BLAST success are presented in Table 1. Our genes of interest were identified and characterised for the three species (Table 2). Most transcripts of interest from the three *Cherax* species were highly conserved at the nucleotide level and had predicted proteins of similar size and sequence composition (Table 2).

**Table 2:** Full length coding sequences of the candidate genes involved in pH balance and osmotic/ionic regulation amplified from the gill transcriptomes of *C. quadricarinatus*, *C. destructor* and *C. cainii*.

		*Redclaw*				*Yabby*				*Marron*			
Genes	Enzymes	Gene ID	Contig length Bp	Protein length (aa)	Accession #	Gene ID	Contig length Bp	Protein length (aa)	Accession #	Gene ID	Contig length Bp	**Protein length** (aa)	Accession #
CAc	Carbonic anhydrase alpha	CqCAc	1,527	271	KM538165	CdCAc	1,589	271	KP299962	CcCAc	2,795	271	KP221715
CAg	Carbonic anhydrase GPI-linked	CqCAg	3,352	310	KM538166	CdCAg	2,465	289	KP299963	CcCAg	4,774	310	KP221716
CAb	Beta carbonic anhydrase	CqCAb	927	257	KM538167	CdCAb	2,459	257	KP299965	CcCAb	4,163	257	KP221717
NKA- α	Sodium/potassium ATPase alpha subunit	CqNKA- α	4,351	1,038	KR270438	CdNKA- α	5,500	1,038	KP299966	CcNKA- α	3,888	1,038	KP221718
NKA- β	Sodium/potassium ATPase beta subunit	CqNKA- β	1,662	316	KP221719	CdNKA- β	1,601	316	KP299967	CcNKA- β	1,551	316	KP221719
HAT-c	V-type H^+^-ATPase Catalytic subunit A	CqHATc	2,626	622	KM538169	CdHATc	2,760	622	KP299968	CcHATc	2,678	622	KP221720
HAT	V-type H^+^-ATPase 116 kda subunit A	CqHAT	3,184	831	KM610229	CdHAT	2,810	838	KP299969	CcHAT	2,908	838	KP221721
AK	Arginine kinase	CqAK	1,432	357	KM610226	CdAK	1,477	357	KP299970	CcAK	1,421	357	KP221722
CRT	Calreticulin	CqCRT	1,722	402	KM538170	CdCRT	1,708	403	KP299971	CcCRT	1,739	402	KP221723
SERCA	Sarco/endoplasmic reticulum Ca^+2^ ATPase	CqSERCA	4,631	1,020	KM538171	CdSERCA	4,344	1,002	KP299972	CcSERCA	6,255	1,020	KP221724
SEPHS	Selenophosphate synthetase	CqSEPHS	2,142	326	KP299975	CdSEPHS	2,160	326	KP299975	CcSEPHS	2,186	326	KP221726
NKCC	Sodium/chloride cotransporter	CqNKCC	4,643	1,061	KP299986	CdNKCC	4,524	909	KP299986	CcNKCC	4,728	1,074	KP221733
NHE3	Sodium/hydrogen exchanger 3	CqNHE3	3,578	943	KM880153	CdNHE3	3,308	943	KP299982	CcNHE3	4,271	986	KP221730
NBC	Sodium/bicarbonate cotransporter isoform 4	CqNBCi4	5,217	1,133	KP299993	CdNBCi4	5,023	1,134	KP299979	CcNBCi4	4,986	1,133	KP299993
NBC	Sodium/bicarbonate cotransporter isoform 5	CqNBCi5	4,855	1,114	KP299994	CdNBCi5	5,188	1,115	KP299980	CcNBCi5	4,915	1,114	KP299994
NCX1	Sodium/calcium exchanger 1	CqNCX1	4,895	846	KM880154	CdNCX1	4,148	849	KP299983	CcNCX1	4,289	848	KP221731

### Sequences alignment, domain analysis and phylogenetic relationships

Multiple protein alignments were performed for CA, NKA and HAT, as these genes are considered important candidate genes responsible for pH balance and osmoregulation in crustaceans.

#### Carbonic anhydrase

Cytoplasmic CA from *C. quadricarinatus*, *C. destructor* and *C. cainii* (CqCAc, CdCAc and CcCAc) encoded a predicted protein of 271 aa (Table 2). No cytoplasmic CA sequences contained a signal peptide; but all predicted proteins had two putative N-glycosylation motifs: (NKS at 122 aa. and NGS at 187 aa. position). Protein alignment revealed that cytoplasmic CAs obtained from *C. quadricarinatus*, *C. destructor* and *C. cainii* had greatest similarity to one another and shared 97–98% identity at the amino acid level (Table S1). BLASTp analysis (homologous protein analysis) showed that the cytoplasmic CAs from *C. quadricarinatus*, *C. destructor* and *C. cainii* were highly conserved with other decapod crustaceans and had greatest similarity with cytoplasmic CA forms from *Penaeus monodon* (75–76% identity, EF672697), *Litopenaeus vannamei* (74–76% identity, HM991703), and *Callinectes sapidus* (72–74% identity, EF375490).

Glycosyl-phosphatidylinositol (GPI)-linked CA forms; CqCAg and CcCAg contained a predicted protein with 310 aa (CdCAg had a partial CD of 289 aa). These predicted protein sequences (CqCAg, CdCAg and CcCAg) all contained a N-terminal signal peptide of 22 amino acids (Met^1^ to Gly^22^). The membrane-associated CA (CAg) obtained from all *Cherax* species shared strong similarity with the CAg forms identified in other crustacean species (for example: 73–74% identity with *C. sapidus,* EF375491; 71–73% identity with *L. vannamei,* JX975725; 72–73% identity with *P. trituberculatus,* JX524150); and the similarity among the three *Cherax* species ranged between 97–99% protein identity Table S1).

The β-CA (257 aa) had moderate to high sequence similarity to β-CA proteins obtained from other arthropod species (for example; 64–65% identity with *Acyrthosiphon pisum,* XP_001943693; 61–63% identity with *D. pulex,* EFX79480 (Colbourne et al., 2011) and62–64% identity with *Nasonia vitripennis,* XP_001606972). *Cherax quadricarinatus*, *C. destructor* and *C. cainii* shared between 98–99% amino acid sequence identity (Table S1).

**Figure 1:**
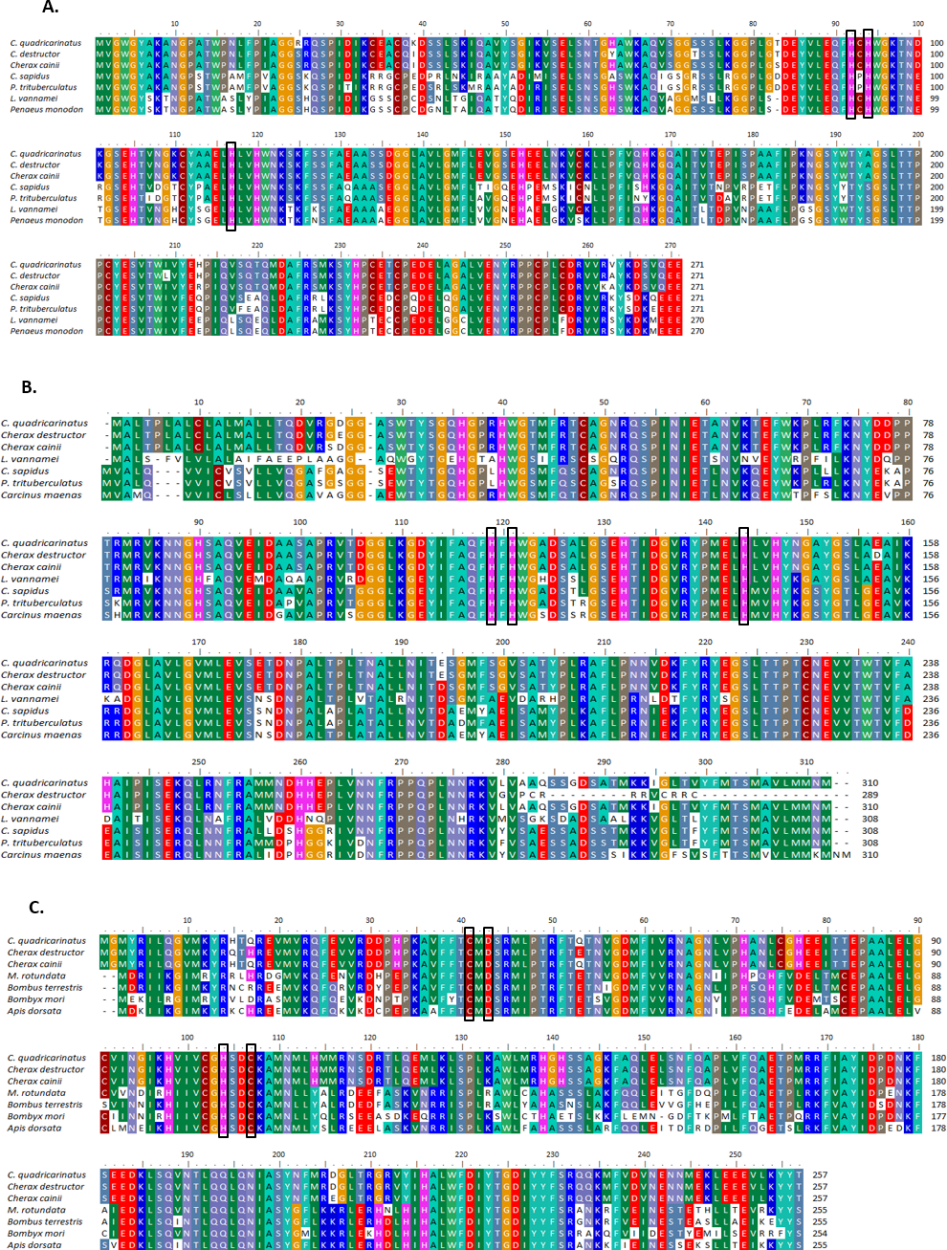
Multiple alignments of amino acid sequences from three Cherax Carbonic anhydrase (CA) with other species. Multiple alignments of amino acid sequences from three *Cherax Carbonic* anhydrase (CA) with other species. Colours indicate the similarity. The black-boxed indicate the predicted active-sites, Zinc-binding residues. (A) Multiple alignment of cytoplasmic CA (B) Multiple alignment of membrane associated CA, and (C) Multiple alignment of β-CA.

**Figure 2:**
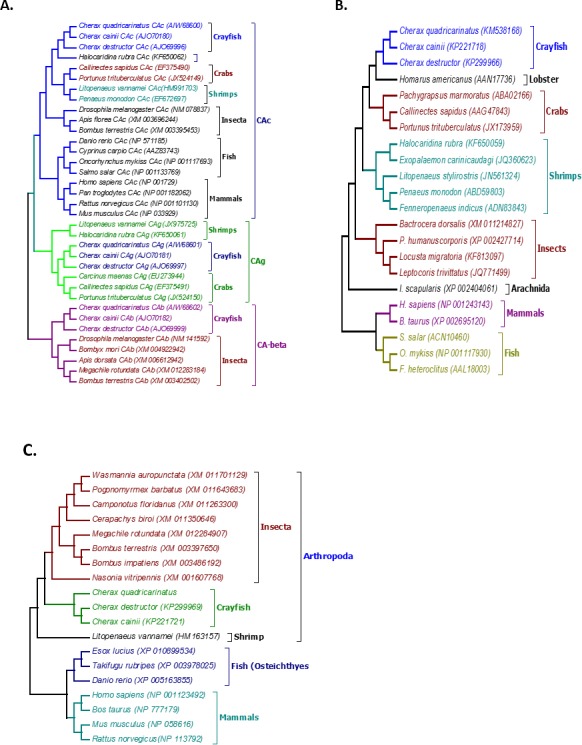
Phylogenetic relationships between the three *Cherax* crayfish with other organisms. Bootstrapped Neighbour-joining tree showing the phylogenetic relationships between the three *Cherax* crayfish with other organisms on the basis of the amino acid sequences. Accession numbers are placed in brackets. (A) Phylogenetic tree of Carbonic Anhydrase proteins (CA) (B) Phylogenetic tree of Na^+^/K^+^-ATPase *α*-subunit (NKA) (C) Phylogenetic tree of V-type-H^+^-ATPase 116 kDa subunit a (HAT-A).

Multiple alignment of all three forms of CAs with their homologous proteins in arthropods revealed that all active sites, including the zinc binding sites and the hydrogen bonding sites around the active sites are conserved (Fig. 1). A Neighbour Joining phylogenetic tree showed that the β-CA proteins formed a separate and well resolved clade from the alpha CA proteins (CAc and CAg). The three *Cherax* species formed a separate group in the β-CA clade that was sister to the same protein from insects. Within the large alpha CA clade, both the CAg and CAc proteins formed distinct well resolved clades. For the CAg clade, the three *Cherax* species formed a monophyletic group that was sister to a group comprised of crabs *Carcinus maenas* (ABX71209, Serrano & Henry, 2008), *Callinectes sapidus* (ABN51214, Serrano, Halanych & Henry, 2007) and *Portunus trituberculatus* (AFV46145, Xu & Liu, 2011); with shrimps being more distantly related *Litopenaeus vannamei* (AGC70493, Liu et al., 2015) and *Halocaridina rubra* (AIM43573, Havird, Santos & Henry, 2014). The CAc clade clustered according to taxonomic affinity where vertebrate species formed a separate group from the arthropod species. Within the arthropod clade, insects species *Drosophila melanogaster* (NP_523561, Hoskins et al., 2007), *Apis florea* (XM_003696244) and *Bombus terrestris* (XP_003395501) formed a separate and well supported group from decapod crustacean species including *Callinectes sapidus,* (ABN51213, Serrano, Halanych & Henry, 2007) and *Portunus trituberculatus* (AFV46144, Xu & Liu, 2011), and shrimp species *Halocaridina rubra* (AIM43574, Havird, Santos & Henry, 2014), *Litopenaeus vannamei* (ADM16544, Liu et al., 2015), *Penaeus monodon* (ABV65904, Pongsomboon et al., 2009) (Fig. 2). The three *Cherax* species clustered together in a discrete group which was sister to the other decapod species.

#### Na^+^/K^+^-ATPase (NKA)

**Figure 3:**
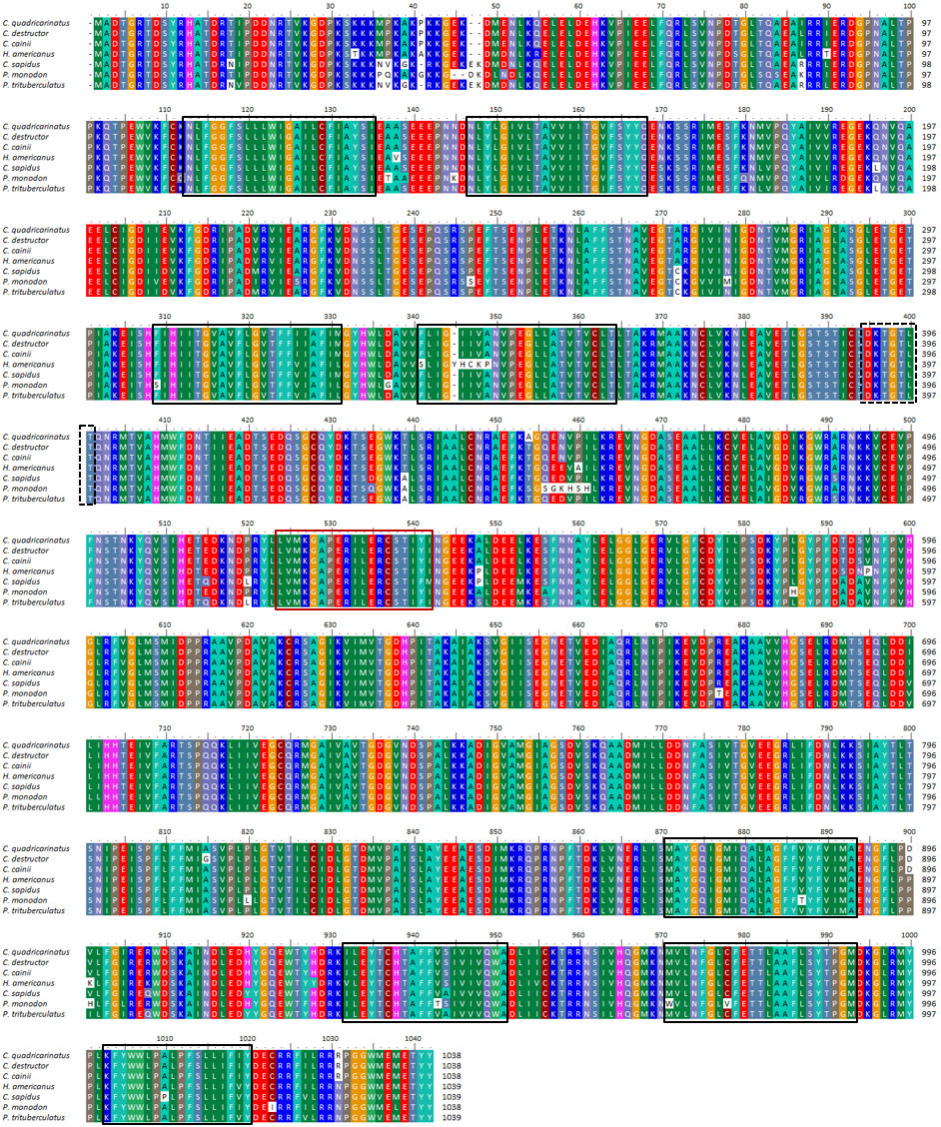
Multiple alignment of three *Cherax* Na^+^/K^+^-ATPase *α*-subunit amino acid sequences with other crustaceans. Multiple alignment of three *Cherax* Na^+^/K^+^-ATPase *α*-subunit amino acid sequences with other crustaceans; *Cherax quadricarinatus* (), *C. destructor* (), *C. cainii* (), *Homarus americanus* (AAN17736), *Callinectes sapidus* (AAG47843), *Paeneus monodon* (ABD59803) and *Portunus trituberculatus* (AGF90965). Putative transmembrane domains are marked by black-boxed; likely ATP-binding site, by red-boxed; and phosphorylation site, by dashed-black boxed.

Full-length nucleotide sequences and predicted aa sequences of the Na^+^/K^+^-ATPase alpha subunit from *C. quadricarinatus*, *C. destructor* and *C. cainii* (CqNKA, CdNKA and CcNKA) were functionally characterised (Table 2). The CqNKA, CdNKA and CcNKA ORFs all produced a predicted protein of 1,038 aa in length. No signal-peptide was present, but eight transmembrane domains were detected through hydrophobicity analysis of the sequences (Fig. 3).

The NKA proteins from *Cherax* species were highly conserved (97–99% aa identity) and showed greatest similarity to NKA from other decapod crustacean species (for example; 96% with *Homarus americanus* (AY140650); 95% with *Eriocheir sinensis* (KC691291); 95% with *C. sapidus* (AF327439) and 93% with *P. monodon* (DQ399797).

Multiple alignment of NKA with the homologous proteins of other crustaceans revealed that all active sites are highly conserved (Fig. 3). The phylogenetic tree based on the *α*-subunit of NKA showed the existence of three distinct groups; arthropods, mammals, and osteichthyes (Fig. 2). The arthropods further clustered into three clades; crustaceans, insects and arachnids. All the NKA *α*-subunit sequence obtained from *C. quadricarinatus*, *C. destructor* and *C. cainii* formed a distinct monophyletic group that was sister to a *Homarus americanus* sequence (AAN17736). Crayfish sequences were more closely related to those of the crab species *Callinectes sapidus* (AAG47843), *Portunus trituberculatus* (JX173959) and *Pachygrapsus marmoratus* (ABA02166) than to shrimp species *Penaeus monodon* (ABD59803), *Fenneropenaeus indicus* (ADN83843), *Halocaridina rubra* (KF650059), *Litopenaeus stylirostris* (JN561324) and *Exopalaemon carinicaudagi* (JQ360623) (Fig. 2).

#### V-type H^+^ATPase (HAT)

In the *Cherax* datasets, multiple H^+^-ATPase (HAT) subunits were identified (Table 2), however, only HAT 116 kDa subunit A was used in phylogenetic analyses. Subunit A was chosen as it is the main catalytic subunit in proton translocation. Translation of ORFS revealed that the predicted proteins from HAT 116 kda subunit A transcripts were the same length across the three *Cherax* species (831 aa, Table 2).

Conserved domains were identified in HAT subunit A including V_ATPase_I domain between 26–818 aa (protein family: pfam01496). Another predicted feature of the HAT-A predicted protein was seven putative transmembrane regions at positions: 398–420, 441–461, 532–550, 567–587, 640–660, 733–751 and 765–785 aa.

**Figure 4:**
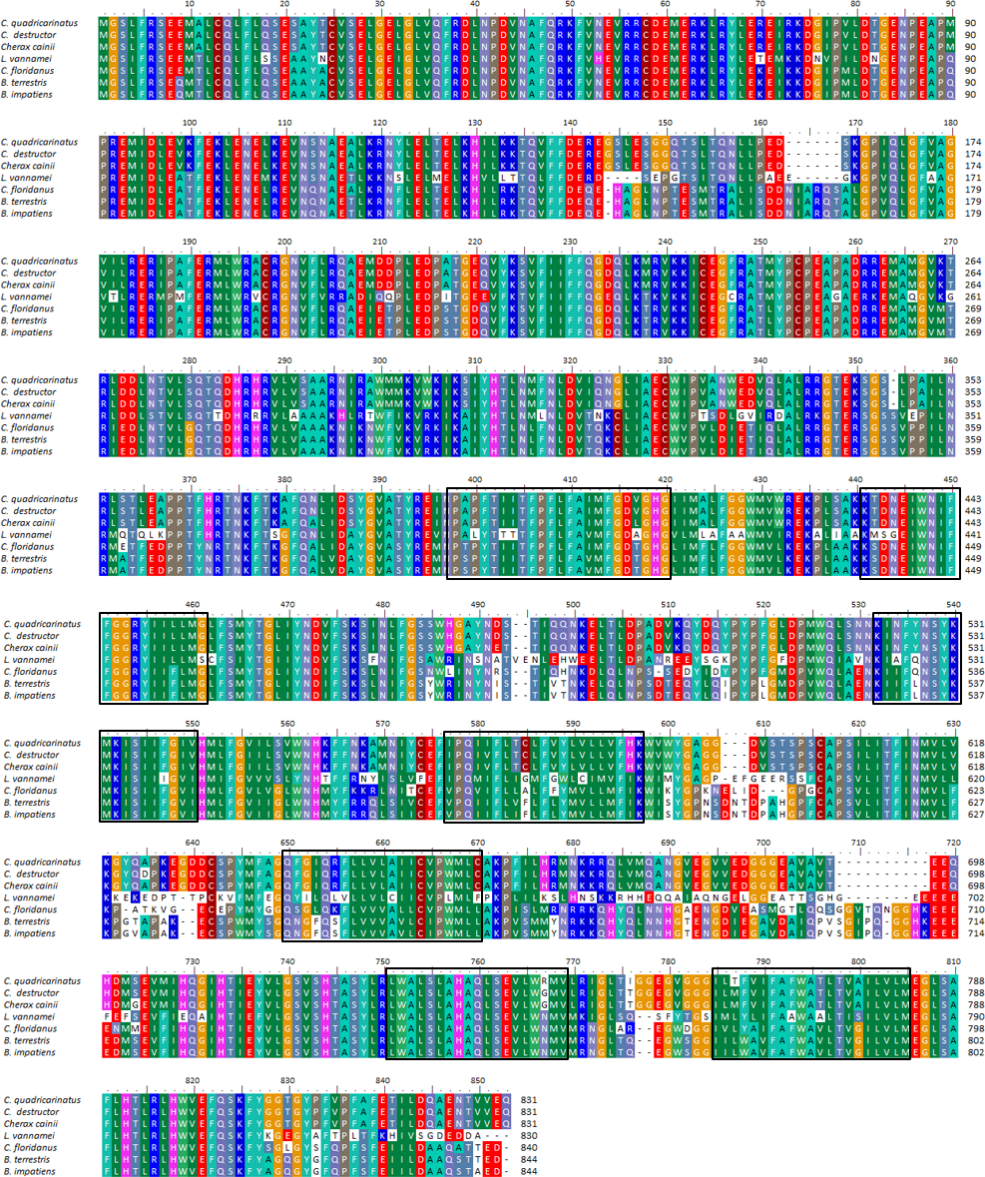
Multiple alignment of three *Cherax* V-type-H^+^-ATPase subunit-a amino acid sequences with other species. Multiple alignment of three *Cherax* V-type- H^+^-ATPase 116 kDa subunit-a amino acid sequences with other closely related species. Putative transmembrane domains are marked by black-boxed.

The HAT-A predicted protein was highly conserved in *C. quadricarinatus*, *C. destructor* and *C. cainii* (97–98% aa identity), as well as across arthropods in general (Fig. 4). The HAT-A from *Cherax* species had the greatest similarity with the HAT-116 kDA from other arthropods (for examples; 63–64% with *L. vannamei* (AEE25939); 71–72% with *B. terrestris* (XP_003397698); 70–71% with *M. rotundata* (XP_012140297); and 70–71% with *C. floridanus* (XP_011261602)).

The phylogenetic tree based on the translated amino acid sequences revealed that V-type-H^+^-ATPase subunit-A formed three distinct groups; arthropods, fish and mammals. Arthropods further clustered into two clades; decapod crustaceans and insects. All crayfish species, *C. quadricarinatus*, *C. destructor* and *C. cainii* formed a monophyletic group that was sister to a group comprised of insects; and were distantly related to the shrimp *L. vannamei*, (AEE25939, Wang et al., 2012). The arthropod clade was distinct from a second well resolved clade of vertebrates (Fig. 2).

#### Other genes

Comparative sequence analyses showed that all genes including Na^+^/K^+^/2Cl^−^ cotransporter (NKCC), Na^+^/Cl^−^/HCO_3_^−^ cotransporter (NBC), Na^+^/H^+^ exchanger 3 (NHE3), Na^+^/Ca^+2^ exchanger 1 (NCX1), Arginine kinase (AK), Sarcoplasmic Ca^+2^-ATase (SERCA) and Calreticulin (CRT) had the putative proteins (the deduced amino acid composition) similar in length and weight across the three species (Table 2). All predicted proteins shared about 97–99% amino acid identity with the corresponding genes across the *Cherax* species (Table S1) and all the functional sites were conserved in the predicted proteins (Table 2 and Table S1).

### Comparative molecular evolutionary analyses

#### Evolutionary analyses of ion transport genes in Cherax sp.

The molecular evolutionary analyses at the codon level between *C. quadricarinatus*, *C. destructor* and *C. cainii* showed that all candidate genes had a number of synonymous substitutions (Table 3). In terms of protein changing mutations, CAc, CAg and β-CA contained 9, 4 and 5 nonsynonymous mutations, respectively (Table 3). The other candidate genes examined in detail, NKA, HAT-A, NKCC, NBC, NHE3, NCX1, AK, SERCA, CRT had 3, 12, 37, 24, 91, 15, 10, 23 and 7 nonsynonymous mutations, respectively (Table 3). Relative evolutionary analysis showed that *C. quadricarinatus*, *C. destructor* and *C. cainii* have had an equal evolutionary rate for all of the candidate genes (the null hypothesis of unequal evolutionary rate was rejected at *p* < 0.05 (Table 3)).

**Table 3:** Molecular evolutionary analyses between three *Cherax* crayfish (*C. quadricarinatus, C. destructor* and *C. cainii*) at amino acid/codon level.

	CAc	CAg	CAb	NKA	HAT-116k	NKCC	NBC	NHE3	NCX1	AK	SERCA	CRT
Total sites/positions in analysis	271	277	257	1,038	831	848	1,145	921	846	357	1,002	402
Identical sites in all three species	262	273	252	1,035	819	811	1,121	830	831	347	979	395
Nonsynonymous sites	9	4	5	3	12	37	24	91	15	10	23	7
Synonymous substitutions at codon	54	57	18	71	85	506	107	143	91	26	55	39
Divergent sites in all three species	0	0	0	0	0	1	1	9	1	1	1	0
Unique differences in *Redclaw*	3	0	0	1	6	11	7	29	7	1	6	1
Unique differences in *Yabby*	3	2	3	2	1	14	7	20	2	3	9	3
Unique differences in *Marron*	3	2	2	0	5	11	9	33	5	5	7	3
Equal evolutionary rate between *Redclaw & Yabby*?	Yes (1.00)^a^	Yes (0.16)	Yes (0.08)	Yes (0.56)	Yes (0.06)	Yes (0.55)	Yes (0.8)	Yes (0.20)	Yes (0.10)	Yes (0.32)	Yes (0.44)	Yes (0.32)
Equal evolutionary rate between *Redclaw &Marron*?	Yes (1.0)	Yes (0.16)	Yes (0.16)	Yes (0.32)	Yes (0.76)	Yes (1)	Yes (0.8)	Yes (0.61)	Yes (0.56)	Yes (0.10)	Yes (0.78)	Yes (0.32)
Equal evolutionary rate between *Yabby & Marron*?	Yes (1.0)	Yes (1.00)	Yes (0.65)	Yes (0.16)	Yes (0.10)	Yes (0.55)	Yes (0.6)	Yes (0.07)	Yes (0.26)	Yes (0.48)	Yes (0.62)	Yes (1.0)
Average evolutionary divergence	0.022	0.010	0.013	0.002	0.010	0.03	0.05	0.072	0.012	0.02	0.016	0.012

**Notes.**

CAc Cytoplasmic carbonic anhydrase CAg GPI-linked carbonic anhydrase CAb Beta carbonic anhydrase NKA Na^+^/K^+^-ATPase alpha subunit HAT-116k Vacuolar type H^+^-ATPase 116 kda NKCC Na^+^/K^+^/2Cl^−^ cotransporter NBC Na^+^/HCO${}_{3}^{-}$cotransporter NHE3 Na^+^/H^+^ exchanger 3 NCX1 Na+/Ca^+2^ exchanger 1 AK Arginine kinase SERCA Sarco/endoplasmic reticulum Ca^+2^-ATPase CRT Calreticulin

a Values in brackets are *p*-values used to reject the null hypothesis of equal evolutionary rate.

#### Evolutionary analyses across crustacean species

Comparative analyses across arthropods, which included mostly crustacean species (around 15 species including three *Cherax* sp.), revealed a large number of non-synonymous mutations in all the sequences (173 in CAc, 128 in CAg, 143 in CAb, 243 in NKA, 355 in HAT-116k, 586 in NKCC, 506 in NBC, 504 in NHE3, 529 in NCX1, 78 in AK, 276 in SERCA and 193 in CRT) (see Table 4). Despite a large number of nonsynonymous mutations, HyPHy analysis (codon-by-codon natural selection estimations) detected that no candidate genes showed patterns of nucleotide variation consistent with the action of natural selection (*p* < 0.05).

**Table 4:** Molecular evolutionary analyses across crustacean species at Amino acid level.

Enzymes	Gene	No. of species/ Sequences in analysis	Total sites in analysis	Total segregating sites	Total Identical sites	Rate of segregation/ site	Nucleotide diversity	In-Del
Cytoplasmic carbonic anhydrase	CAc	15	265	173	92	0.65283	0.38225	0
GPI-linked carbonic anhydrase	CAg	8	287	128	159	0.445993	0.24029	0
Beta carbonic anhydrase	CAb	16	254	143	111	0.562992	0.2691	0
Na^+^/K^+^-ATPase alpha subunit	NKA	16	1,001	243	758	0.242757	0.08577	0
Vacuolar type H^+^-ATPase 116 kda	HAT-116k	15	803	355	448	0.442092	0.19255	0
Na^+^/K^+^/2Cl^−^ cotransporter	NKCC	16	838	586	252	0.699284	0.39172	0
Na^+^/HCO${}_{3}^{-}$ cotransporter	NBC	16	1,033	506	527	0.489835	0.23662	0
Na^+^/H^+^ exchanger 3	NHE3	15	843	504	339	0.597865	0.25904	0
Na+/Ca^+2^ exchanger 1	NCX1	15	793	529	264	0.667087	0.28318	0
Arginine kinase	AK	15	355	78	277	0.219718	0.07329	0
Sarco/endoplasmic reticulum Ca^+2^-ATPase	SERCA	15	989	276	713	0.27907	0.12366	0
Calreticulin	CRT	15	396	193	203	0.487374	0.212	0

### Tissue-specific expression analyses

**Figure 5:**
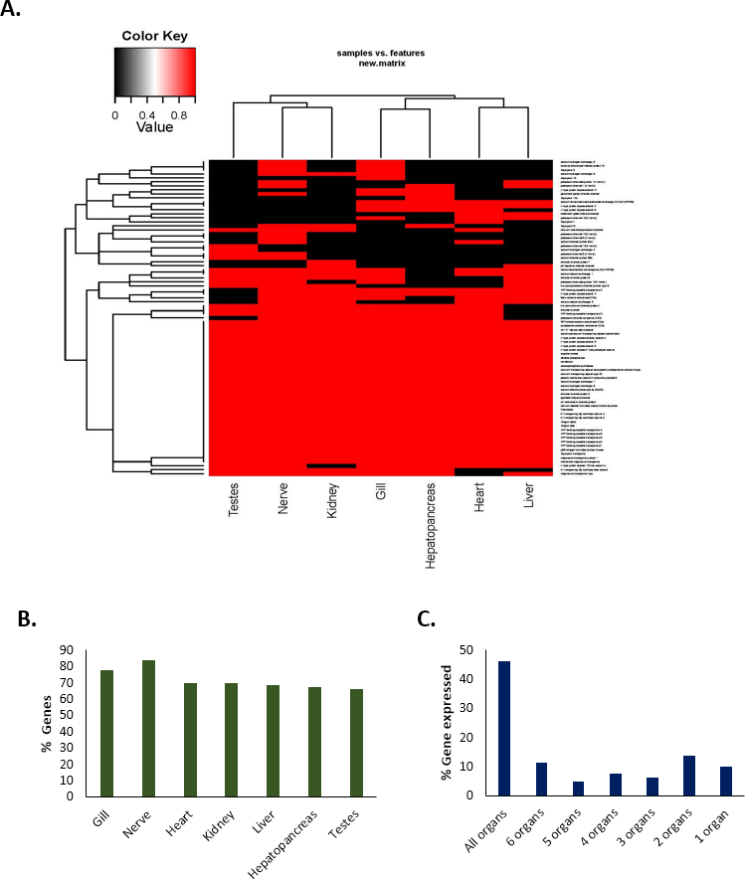
Tissue-specific expression/occurrences of important genes associated with pH balance or ion-regulation in Redclaw crayfish (*Cherax quadricarinatus*). (A) Presence-absence heat-map (B) Tissue-specific percentage of genes expressed (C) Maximum types of organs with % of genes expressed therein.

The expression of a total of 80 important genes that are directly or indirectly associated with either acid–base balance or osmotic/ionic regulation was investigated in seven different tissue types (gills, hepatopancreas, heart, kidney, liver, nerve and testes) in *C. quadricarinatus* (Table S2). The results showed that 46% of the genes (37) were expressed in all types of tissues; 11% (nine genes) in six tissue types, 5% (four genes) in five tissue types, 8% (six genes) in four tissue types; 6% (five genes) in three tissue types; 14% (11 genes) in two tissue types (Fig. 5). Approximately 10% of the genes (eight) were expressed in only one tissue type, i.e., they were unique in that particular type of tissue. The highest number of genes were observed in the nerve (84%, 67 genes) followed by the gills (78%, 62 genes). The percentage of genes expressed in other tissues are 70% for heart (56 genes), 70% for kidney (56 genes), 69% for liver (55 genes), 67.5% for hepatopancreas (54 genes), and 66% for testes (53 genes) (Fig. 5).

## Discussion

Overall this study has demonstrated that most of the candidate genes involved in systemic acid–base balance and/or ion transport are highly conserved in the genus *Cherax* and in crustacean species in general. While these genes showed little variation across the three target species, we found no evidence of purifying selection shaping nucleotide variation in any of the genes.

### Comparative molecular analyses

#### Carbonic anhydrase

Comparative analysis across the three *Cherax* species showed that both cytoplasmic and membrane linked CA were highly conserved, which may be explained by the conserved function of CA. The molecular structure of CA comprises four primary components: the Zn binding sites, the substrate association pocket, the threonine-199 loop and the proton shuttling mechanism (Christianson & Alexander, 1989; Merz, 1990; Krebs et al., 1993). In our study, we did not find any mutations in these important functional sites at the amino acid level in both cytoplasmic and membrane-associated isoforms of CA across the *Cherax* species. We did, however, find amino acid replacements outside of these functional sites and these may be important for adaptation to different pH environments in *Cherax* species.

Previous research has shown that many *Cherax* species, including *C. quadricarinatus*, *C. destructor* and *C. cainii*, naturally occur in locations with water of different pH levels (Macaranas et al., 1995; Bryant & Papas, 2007; Baker, De Bruyn & Mather (2008). Our recent study shows that cytoplasmic and membrane-associated isoforms of carbonic anhydrase gene expressed differentially at different pH levels (Ali et al., 2015b). Therefore, amino acid replacements in cytoplasmic and membrane linked CA may be associated with the differences in water chemistry experienced by different species.

#### Na^+^/K^+^-ATPase

All the expected features of NKA were highly conserved in the three *Cherax* species, including eight transmembrane domains (Mitsunaga-Nakatsubo et al., 1996), the putative ATP-binding site (Horisberger et al., 1991) and a phosphorylation site. In fact, only two conservative amino acid replacements were observed among the three *Cherax* species, an alanine to glycine replacement in *C. destructor* and an alanine to threonine replacement in *C*. *quadricarinatus*. Given that these *Cherax* species are naturally distributed in environments with diverse salinity and pH ranges (Bryant & Papas, 2007), you might expect to find more amino acid replacements in this gene. The lack of variation at the amino acid level may indicate other mechanisms are important for the response to salinity and pH changes in *Cherax* species. Changes in the level of expression of NKA associated with salinity or pH changes may be a plausible hypothesis for this lack of amino acid variation among *Cherax* species. A number of studies have demonstrated that NKA expression is strongly induced by changes in salinity and/or pH in the gills of decapod crustaceans lending support to this hypothesis (Luquet et al., 2005; Serrano & Henry, 2008; Wang et al., 2012; Han et al., 2015; Leone et al., in press; Li et al., 2015). This hypothesis needs to be further tested, however, before it can be supported.

#### H^+^-ATPase

Inter-species evolutionary analyses between the three *Cherax* crayfish and other arthropod species showed that V-H^+^-ATPase subunit A (HAT-A) was largely conserved at the protein sequence level, but had patterns of nucleotide variation consistent with neutral evolution. The small amount of amino acid variation found at this enzyme in *Cherax* species is probably because of its conserved function, as it is one of key enzymes via which many crustaceans maintain their internal acid–base balance (Kitagawa et al., 2008; Faleiros et al., 2010; Lee et al., 2011; Muench, Trinick & Harrison, 2011; Towle, Henry & Terwilliger, 2011; Wang et al., 2012; Boudour-Boucheker et al., 2014; Marshansky, Rubinstein & Grüber, 2014; Lucena et al., 2015; Rawson et al., 2015). As the maintenance of acid–base balance is very important in aquatic crustaceans, amino acid replacements in important functional domains might have a deleterious effect on the function and activity of this enzyme. Alternatively, amino acid replacements in V-H^+^-ATPase subunit A that increase its activity under different pH conditions may be beneficial to the different *Cherax* species.

#### Other genes

The comparative molecular analyses showed that most other genes including Na^+^/K^+^/2Cl^−^ cotransporter (NKCC), Na^+^/Cl^−^/HCO_3_^−^ cotransporter (NBC), Na^+^/H^+^ exchanger 3 (NHE3), Na^+^/Ca^+2^ exchanger 1 (NCX1), Arginine kinase (AK), Sarcoplasmic Ca^+2^-ATase (SERCA) and Calreticulin (CRT) were conserved across the three *Cherax* crayfish (Tables 3 and 4). The underlying reason may be that the these genes encode a set of enzymes that are actively or passively associated with the fundamental and common physiological processes of ion-regulation in crustaceans (Lucu, 1989; Onken, Graszynski & Zeiske, 1991; Onken, Tresguerres & Luquet, 2003; Ahearn, Mandal & Mandal, 2004; Serrano, Halanych & Henry, 2007; Mandal et al., 2009; Lv et al., 2015; Ren et al., 2015; Xu et al., 2015). The high level of amino acid conservation in these predicted proteins may be attributed to the fact that they are found in important biochemical pathways that influence systemic acid–base balance and/or ion transport (Uda et al., 2006; Hwang, Lee & Lin, 2011; Hiroi & McCormick, 2012; McNamara & Faria, 2012). These genes have previously been demonstrated to function as important enzymes involved in osmoregulatory organs such as gills and epipodites in a number of crustaceans (Towle & Weihrauch, 2001; Weihrauch et al., 2004; Freire, Onken & McNamara, 2008; Mandal et al., 2009). In fact, gene expression data for Na^+^/K ^+^/2Cl^−^ cotransporter (Luquet et al., 2005; Havird, Henry & Wilson, 2013) and Calreticulin (Luana et al., 2007; Lv et al., 2015; Xu et al., 2015) show that they are induced under different ionic conditions and arginine kinase has also been shown to be induced under different pH and salinity conditions (Serrano, Halanych & Henry, 2007; Xie et al., 2014; Ali et al., 2015b).

### Tissue-specific expression analyses

Tissue-specific expression of the candidate genes involved in systemic acid–base balance revealed that most of the major genes involved in this process including carbonic anhydrase (CA), Na^+^/K^+^-ATPase (NKA), Vacuolar type H^+^-ATPase (HAT), Na^+^/H^+^ exchanger (NHE), Na^+^/K^+^/2Cl^−^ cotransporter (NKCC), Na^+^/Cl^−^/HCO${}_{3}^{-}$cotransporter, Arginine kinase (AK) and Sarco/endoplasmic reticulum Ca^+2^-ATPase (SERCA) were expressed in most tissue types. This indicates that the majority of ion transport genes are expressed in most tissue types in freshwater crayfish and is something that needs to be taken into account in future gene expression studies of osmoregulation genes in specific tissues such as gills. As all the major genes were expressed in tissues other than gills, it implies an important physiological role for these genes outside of osmoregulation. For example Na^+^/K^+^-ATPase, an important osmoregulatory gene, has other important functions including as a signal transducer/integrator that regulates the mitogen activated stress kinase pathway, intracellular calcium concentrations and reactive oxygen species (Xie & Askari, 2002; Yuan et al., 2005; Bhavsar et al., 2014). As many osmoregulation pH balance genes potentially encode for multifunctional proteins that are required to perform important functions their expression across multiple tissue types is probably not surprising.

The two tissue types with highest number of candidate genes expressed were the gills and nervous system. A large number of genes involved in acid–base balance and osmic-/ionic-regulation expressed in the gills was expected based on previous research in decapod crustaceans (Freire, Onken & McNamara, 2008; Romano & Zeng, 2012). The largest number of ion transport and pH balance candidate genes were expressed in nervous tissue and this data is supported by recent research on the American lobster *Homarus americanus* (McGrath et al., 2016). In this paper the authors found that a large number of ion channel gene families were over expressed in nerve tissues relative to heart and abdominal muscle. This pattern is not surprising as many ion transport related genes are highly expressed in nervous tissue as they play an important role in generating action potential (Fry & Jabr, 2010; Benarroch, 2011; Deval & Lingueglia, 2015; Hertz et al., 2015; Shrivastava et al., 2015; Wu et al., 2015; Zhang et al., 2015).

## Conclusions

We analysed the molecular differences at amino acid and nucleotide levels in most of the major genes involved in acid–base balance and osmotic/ionic regulation in three freshwater crayfish of the genus *Cherax*. The majority of these genes were expressed across most tissue types and were highly conserved at the amino-acid level. These findings indicate that these genes are important and probably have diverse functions related to ion exchange and pH balance across different tissues in freshwater crayfish.

##  Supplemental Information

10.7717/peerj.3623/supp-1Table S1Identity matrix across species based on amino acid compositionsIdentity matrix based on amino acid composition between three Cherax species (Cherax quadricarnatus, Cherax cainii and Cherax destructor) and other crustaceans. The first row and first column indicate species. Values in the cells indicate identity in percentage. CAc, Cytoplasmic carbonic anhydrase; CAg, GPI-linked carbonic anhydrase; CAb, Beta carbonic anhydrase; NKA, Na^+^/K^+^-ATPase alpha subunit; HAT, Vacuolar type H^+^-ATPase 116 kda; NKCC, Na^+^/K^+^/2Cl^−^ cotransporter; NBC, Na^+^/HCO${}_{3}^{-}$ cotransporter; NHE, Na^+^/H^+^ exchanger 3; NCX, Na^+^/Ca^+2^ exchanger 1; AK, Arginine kinase; and CRT, Calreticulin.

10.7717/peerj.3623/supp-2 Table S2 Tissue-specific expression of major pH- and osmoregulatory genes in Redclaw crayfish *Cherax quadricarinatus* Information on whether the genes are present or not in particular tissue. ‘p’ in the celll indicates present and ‘a’ indicates absent. A total of eighty (80) most important genes associated with pH balance and osmo-regulation were observed.

10.7717/peerj.3623/supp-3 File S1 Contig information—Gills Annotated information of all the assembled contings obtained from Gills of freshwater crayfish Cherax quadricarinatus. Reads which met our quality criteria (*Q* > 30, N bases <1%) were accepted for assembly. The filtered reads were assembled with the Trinity short read *de novo* assembler using the stranded option for Illumina data (Version 2014-04-13p1) and the unstranded option for Ion Torrent data. Assembled data were used as BLASTx queries against the NCBI NR protein database using BLAST + (Version 2.2.29) with a stringency of 10 × *e*−^5^. First row contains information on: number of original contigs, sequence length (contig length bp), seq. description (gene names), number of HITs and mean *e*-values (stringency).

10.7717/peerj.3623/supp-4 File S2 Contig information—Heart Annotated information of all the assembled contings obtained from Heart of freshwater crayfish Cherax quadricarinatus. Reads which met our quality criteria (*Q* > 30, N bases <1%) were accepted for assembly. The filtered reads were assembled with the Trinity short read *de novo* assembler using the stranded option for Illumina data (Version 2014-04-13p1) and the unstranded option for Ion Torrent data. Assembled data were used as BLASTx queries against the NCBI NR protein database using BLAST + (Version 2.2.29) with a stringency of 10 × *e*−^5^. First row contains information on: number of original contigs, sequence length (contig length bp), seq. description (gene names), number of HITs and mean *e*-values (stringency).

10.7717/peerj.3623/supp-5 File S3 Contig information—Kidney Annotated information of all the assembled contings obtained from Kidneys of freshwater crayfish Cherax quadricarinatus. Reads which met our quality criteria (*Q* > 30, N bases <1%) were accepted for assembly. The filtered reads were assembled with the Trinity short read *de novo* assembler using the stranded option for Illumina data (Version 2014-04-13p1) and the unstranded option for Ion Torrent data. Assembled data were used as BLASTx queries against the NCBI NR protein database using BLAST + (Version 2.2.29) with a stringency of 10 × *e*−^5^. First row contains information on: number of original contigs, sequence length (contig length bp), seq. description (gene names), number of HITs and mean *e*-values (stringency).

10.7717/peerj.3623/supp-6 File S4 Contig information—Liver Annotated information of all the assembled contings obtained from liver of freshwater crayfish Cherax quadricarinatus. Reads which met our quality criteria (*Q* > 30, N bases <1%) were accepted for assembly. The filtered reads were assembled with the Trinity short read *de novo* assembler using the stranded option for Illumina data (Version 2014-04-13p1) and the unstranded option for Ion Torrent data. Assembled data were used as BLASTx queries against the NCBI NR protein database using BLAST + (Version 2.2.29) with a stringency of 10 × *e*−^5^. First row contains information on: number of original contigs, sequence length (contig length bp), seq. description (gene names), number of HITs and mean *e*-values (stringency).

10.7717/peerj.3623/supp-7 File S5 Contig information—Nerve Annotated information of all the assembled contings obtained from nerves of freshwater crayfish Cherax quadricarinatus. Reads which met our quality criteria (*Q* > 30, N bases <1%) were accepted for assembly. The filtered reads were assembled with the Trinity short read *de novo* assembler using the stranded option for Illumina data (Version 2014-04-13p1) and the unstranded option for Ion Torrent data. Assembled data were used as BLASTx queries against the NCBI NR protein database using BLAST + (Version 2.2.29) with a stringency of 10 × *e*−^5^. First row contains information on: number of original contigs, sequence length (contig length bp), seq. description (gene names), number of HITs and mean *e*-values (stringency).

10.7717/peerj.3623/supp-8 File S6 Contig information—Testes Annotated information of all the assembled contings obtained from Testes of freshwater crayfish Cherax quadricarinatus. Reads which met our quality criteria (*Q* > 30, N bases <1%) were accepted for assembly. The filtered reads were assembled with the Trinity short read *de novo* assembler using the stranded option for Illumina data (Version 2014-04-13p1) and the unstranded option for Ion Torrent data. Assembled data were used as BLASTx queries against the NCBI NR protein database using BLAST+ (Version 2.2.29) with a stringency of 10 × *e*−^5^. First row contains information on: number of original contigs, sequence length (contig length bp), seq. description (gene names), number of HITs and mean *e*-values (stringency).

10.7717/peerj.3623/supp-9 File S7 Contig information—Hepatopancreas Annotated information of all the assembled contings obtained from Hepatopancreas of freshwater crayfish Cherax quadricarinatus. Reads which met our quality criteria (*Q* > 30, N bases <1%) were accepted for assembly. The filtered reads were assembled with the Trinity short read *de novo* assembler using the stranded option for Illumina data (Version 2014-04-13p1) and the unstranded option for Ion Torrent data. Assembled data were used as BLASTx queries against the NCBI NR protein database using BLAST + (Version 2.2.29) with a stringency of 10 × *e*−^5^. First row contains information on: number of original contigs, sequence length (contig length bp), seq. description (gene names), number of HITs and mean *e*-values (stringency).

## Additional Information and Declarations

### Data Availability

The following information was supplied regarding data availability: All raw sequence data was deposited in the Sequence Read Archive under the Bio-project numbers PRJNA275170, PRJNA275038 and PRJNA275165. Information relating assembled contigs and annotations are provided in the Supplemental Files.

## References

[ref-1] Ahearn GA, Mandal PK, Mandal A (2004). Calcium regulation in crustaceans during the molt cycle: a review and update. Comparative Biochemistry and Physiology Part A: Molecular & Integrative Physiology.

[ref-2] Ali MY, Pavasovic A, Amin S, Mather PB, Prentis PJ (2015a). Comparative analysis of gill transcriptomes of two freshwater crayfish, *Cherax cainii* and *C. destructor*. Marine Genomics.

[ref-3] Ali MY, Pavasovic A, Mather PB, Prentis PJ (2015b). Analysis, characterisation and expression of gill-expressed carbonic anhydrase genes in the freshwater crayfish Cherax quadricarinatus. Gene.

[ref-4] Amin S, Prentis PJ, Gilding EK, Pavasovic A (2014). Assembly and annotation of a non-model gastropod (Nerita melanotragus) transcriptome: a comparison of De novo assemblers. BMC Research Notes.

[ref-5] Baker N, De Bruyn M, Mather PB (2008). Patterns of molecular diversity in wild stocks of the redclaw crayfish (*Cherax quadricarinatus*) from northern Australia and Papua New Guinea: impacts of Plio-Pleistocene landscape evolution. Freshwater Biology.

[ref-6] Benarroch EE (2011). Na^+^, K^+^-ATPase Functions in the nervous system and involvement in neurologic disease. Neurology.

[ref-7] Bhavsar SK, Hosseinzadeh Z, Brenner D, Honisch S, Jilani K, Liu G, Szteyn K, Sopjani M, Mak TW, Shumilina E, Lang F (2014). Energy-sensitive regulation of Na^+^/K^+^-ATPase by Janus kinase 2. American Journal of Physiology-Cell Physiology.

[ref-8] Bolger AM, Lohse M, Usadel B (2014). Trimmomatic: a flexible trimmer for Illumina Sequence Data. Bioinformatics.

[ref-9] Boudour-Boucheker N, Boulo V, Charmantier-Daures M, Grousset E, Anger K, Charmantier G, Lorin-Nebel C (2014). Differential distribution of V-type H+-ATPase and Na^+^/K^+^-ATPase in the branchial chamber of the palaemonid shrimp Macrobrachium amazonicum. Cell and Tissue Research.

[ref-10] Bryant D, Papas P (2007). Marron Cherax Cainii (Austin) in Victoria: a literature review.

[ref-11] Camacho C, Coulouris G, Avagyan V, Ma N, Papadopoulos J, Bealer K, Madden T (2009). BLAST+: architecture and applications. BMC Bioinformatics.

[ref-12] Chaudhari A, Gireesh-Babu P, Tripathi G, Sabnis S, Dhamotharan K, Vardarajan R, Kumari K, Dasgupta S, Rajendran K (2015). Expression studies on NA^+^/K^+^-ATPase in gills of Penaeus monodon (Fabricius) acclimated to different salinities. Indian Journal of Experimental Biology.

[ref-13] Christianson DW, Alexander RS (1989). Carboxylate-histidine-zinc interactions in protein structure and function. Journal of the American Chemical Society.

[ref-14] Colbourne JK, Pfrender ME, Gilbert D, Thomas WK, Tucker A, Oakley TH, Tokishita S, Aerts A, Arnold GJ, Basu MK, Bauer DJ, Cáceres CE, Carmel L, Casola C, Choi J-H, Detter JC, Dong Q, Dusheyko S, Eads BD, Fröhlich T, Geiler-Samerotte KA, Gerlach D, Hatcher P, Jogdeo S, Krijgsveld J, Kriventseva EV, Kültz D, Laforsch C, Lindquist E, Lopez J, Manak JR, Muller J, Pangilinan J, Patwardhan RP, Pitluck S, Pritham EJ, Rechtsteiner A, Rho M, Rogozin IB, Sakarya O, Salamov A, Schaack S, Shapiro H, Shiga Y, Skalitzky C, Smith Z, Souvorov A, Sung W, Tang Z, Tsuchiya D, Tu H, Vos H, Wang M, Wolf YI, Yamagata H, Yamada T, Ye Y, Shaw JR, Andrews J, Crease TJ, Tang H, Lucas SM, Robertson HM, Bork P, Koonin EV, Zdobnov EM, Grigoriev IV, Lynch M, Boore JL (2011). The ecoresponsive genome of *Daphnia pulex*. Science.

[ref-15] Conesa A, Götz S, García-Gómez JM, Terol J, Talón M, Robles M (2005). Blast2GO: a universal tool for annotation, visualization and analysis in functional genomics research. Bioinformatics.

[ref-16] Deval E, Lingueglia E (2015). Acid-sensing ion channels and nociception in the peripheral and central nervous systems. Neuropharmacology.

[ref-17] Duan Y, Liu P, Li J, Wang Y, Li J, Chen P (2014). Molecular responses of calreticulin gene to Vibrio anguillarum and WSSV challenge in the ridgetail white prawn Exopalaemon carinicauda. Fish & Shellfish Immunology.

[ref-18] Eissa N, Wang H-P (2014). Transcriptional stress responses to environmental and husbandry stressors in aquaculture species. Reviews in Aquaculture.

[ref-19] Faleiros RO, Goldman MHS, Furriel RPM, McNamara JC (2010). Differential adjustment in gill Na^+^/K^+^- and V-ATPase activities and transporter mRNA expression during osmoregulatory acclimation in the cinnamon shrimp *Macrobrachium amazonicum* (Decapoda, Palaemonidae). The Journal of Experimental Biology.

[ref-20] Flik G, Haond C (2000). Na, K-ATPase, Ca-ATPase and Na/Ca-exchange activities in gills, epipodites and branchiostegite of European lobster, Homarus gammarus: effects of exposure to dilute seawater. Journal of Experimental Biology.

[ref-21] Flik G, Verbost PM, Atsma W, Lucu C (1994). Calcium transport in gill plasma membranes of the crab Carcinus maenas: evidence for carriers driven by ATP and a Na^+^ gradient. The Journal of Experimental Biology.

[ref-22] Freire CA, Onken H, McNamara JC (2008). A structure-function analysis of ion transport in crustacean gills and excretory organs. Comparative Biochemistry and Physiology Part A: Comparative Physiology.

[ref-23] Fry CH, Jabr RI (2010). The action potential and nervous conduction. Surgery.

[ref-24] Haas BJ, Papanicolaou A, Yassour M, Grabherr M, Blood PD, Bowden J, Couger MB, Eccles D, Li B, Lieber M, MacManes MD, Ott M, Orvis J, Pochet N, Strozzi F, Weeks N, Westerman R, William T, Dewey CN, Henschel R, LeDuc RD, Friedman N, Regev A (2013). De novo transcript sequence reconstruction from RNA-seq using the Trinity platform for reference generation and analysis. Nature Protocols.

[ref-25] Hall TA (1999). BioEdit: a user-friendly biological sequence alignment editor and analysis program for Windows 95/98/NT. Nucleic Acids Symposium Series.

[ref-26] Han X, Liu P, Gao B, Wang H, Duan Y, Xu W, Chen P (2015). Na^+^/K^+^-ATPase *α*-subunit in swimming crab Portunus trituberculatus: molecular cloning, characterization, and expression under low salinity stress. Chinese Journal of Oceanology and Limnology.

[ref-27] Havird JC, Henry RP, Wilson AE (2013). Altered expression of Na^+^/K^+^-ATPase and other osmoregulatory genes in the gills of euryhaline animals in response to salinity transfer: a meta-analysis of 59 quantitative PCR studies over 10 years. Comparative Biochemistry and Physiology Part D: Genomics and Proteomics.

[ref-28] Havird JC, Santos SR, Henry RP (2014). Osmoregulation in the Hawaiian anchialine shrimp Halocaridina rubra (Crustacea: Atyidae): expression of ion transporters, mitochondria-rich cell proliferation and hemolymph osmolality during salinity transfers. The Journal of Experimental Biology.

[ref-29] Henry RP, Lucu C, Onken H, Weihrauch D (2012). Multiple functions of the crustacean gill: osmotic/ionic regulation, acid–base balance, ammonia excretion, and bioaccumulation of toxic metals. Frontiers in Physiology.

[ref-30] Hertz L, Song D, Xu J, Peng L, Gibbs ME (2015). Role of the astrocytic Na^+^, K^+^-ATPase in K^+^ homeostasis in brain: K^+^ uptake, signaling pathways and substrate utilization. Neurochemical Research.

[ref-31] Hiroi J, McCormick SD (2012). New insights into gill ionocyte and ion transporter function in euryhaline and diadromous fish. Respiratory Physiology & Neurobiology.

[ref-32] Horisberger J, Lemas V, Kraehenbuhl J, Rossier B (1991). Structure-function relationship of Na, K-ATPase. Annual Review of Physiology.

[ref-33] Hoskins RA, Carlson JW, Kennedy C, Acevedo D, Evans-Holm M, Frise E, Wan KH, Park S, Mendez-Lago M, Rossi F, Villasante A, Dimitri P, Karpen GH, Celniker SE (2007). Sequence finishing and mapping of drosophila melanogaster heterochromatin. Science.

[ref-34] Huang Y, Niu B, Gao Y, Fu L, Li W (2010). CD-HIT Suite: a web server for clustering and comparing biological sequences. Bioinformatics.

[ref-35] Hwang P-P, Lee T-H, Lin L-Y (2011). Ion regulation in fish gills: recent progress in the cellular and molecular mechanisms. American Journal of Physiology-Regulatory, Integrative and Comparative Physiology.

[ref-36] Kearse M, Moir R, Wilson A, Stones-Havas S, Cheung M, Sturrock S, Buxton S, Cooper A, Markowitz S, Duran C, Thierer T, Ashton B, Meintjes P, Drummond A (2012). Geneious basic: an integrated and extendable desktop software platform for the organization and analysis of sequence data. Bioinformatics.

[ref-37] Kitagawa N, Mazon H, Heck AJR, Wilkens S (2008). Stoichiometry of the peripheral stalk subunits E and G of yeast V1-ATPase determined by mass spectrometry. Journal of Biological Chemistry.

[ref-38] Kosakovsky PSL, Frost SDW (2005). Not so different after all: a comparison of methods for detecting amino acid sites under selection. Molecular Biology and Evolution.

[ref-39] Krebs JF, Rana F, Dluhy RA, Fierke CA (1993). Kinetic and spectroscopic studies of hydrophilic amino acid substitutions in the hydrophobic pocket of human carbonic anhydrase II. Biochemistry.

[ref-40] Larkin M, Blackshields G, Brown N, Chenna R, McGettigan P, McWilliam H, Valentin F, Wallace I, Wilm A, Lopez R, Thompson J, Gibson T, Higgins D (2007). Clustal W and Clustal X version 2.0. Bioinformatics.

[ref-41] Lee CE, Kiergaard M, Gelembiuk GW, Eads BD, Posavi M (2011). Pumping ions: rapid parallel evolution of ionic regulation following habitat invasions. Evolution.

[ref-42] Leone FA, Garçon DP, Lucena MN, Faleiros RO, Azevedo SV, Pinto MR, McNamara JC (2015). Gill-specific (Na^+^, K^+^) -ATPase activity and *α*-subunit mRNA expression during low-salinity acclimation of the ornate blue crab Callinectes ornatus (Decapoda, Brachyura). Comparative Biochemistry and Physiology Part B: Biochemistry and Molecular Biology.

[ref-43] Li J, Ma P, Liu P, Chen P, Li J (2015). The roles of Na^+^/K^+^-ATPase *α*-subunit gene from the ridgetail white prawn Exopalaemon carinicauda in response to salinity stresses. Fish & Shellfish Immunology.

[ref-44] Liu M, Liu S, Hu Y, Pan L (2015). Cloning and expression analysis of two carbonic anhydrase genes in white shrimp Litopenaeus vannamei, induced by pH and salinity stresses. Aquaculture.

[ref-45] Luana W, Li F, Wang B, Zhang X, Liu Y, Xiang J (2007). Molecular characteristics and expression analysis of calreticulin in Chinese shrimp Fenneropenaeus chinensis. Comparative Biochemistry and Physiology Part B: Biochemistry and Molecular Biology.

[ref-46] Lucena MN, Pinto MR, Garçon DP, McNamara JC, Leone FA (2015). A kinetic characterization of the gill V(H+)-ATPase in juvenile and adult Macrobrachium amazonicum, a diadromous palaemonid shrimp. Comparative Biochemistry and Physiology Part B: Biochemistry and Molecular Biology.

[ref-47] Lucu Č (1989). Evidence for Cl− exchangers in perfused Carcinus gills. Comparative Biochemistry and Physiology Part A: Physiology.

[ref-48] Lucu Č, Flik G (1999). Na^+^-K^+^-ATPase and Na^+^/Ca2+ exchange activities in gills of hyperregulating Carcinus maenas. American Journal of Physiology-Regulatory, Integrative and Comparative Physiology.

[ref-49] Luquet CM, Weihrauch D, Senek M, Towle DW (2005). Induction of branchial ion transporter mRNA expression during acclimation to salinity change in the euryhaline crab *Chasmagnathus granulatus*. Journal of Experimental Biology.

[ref-50] Lv J, Wang Y, Zhang D, Gao B, Liu P, Li J (2015). Cloning and characterization of calreticulin and its association with salinity stress in P. trituberculatus. Cell Stress and Chaperones.

[ref-51] Macaranas JM, Mather PB, Hoeben P, Capra MF (1995). Assessment of genetic variation in wild populations of the redclaw crayfish (*Cherax quadricarinatus*, von Martens 1868) by means of allozyme and RAPD-PCR markers. Marine and Freshwater Research.

[ref-52] Mandal A, Arunachalam SC, Meleshkevitch EA, Mandal PK, Boudko DY, Ahearn GA (2009). Cloning of sarco-endoplasmic reticulum Ca2^+^-ATPase (SERCA) from Caribbean spiny lobster Panulirus argus. Journal of Comparative Physiology B.

[ref-53] Marchler-Bauer A, Derbyshire MK, Gonzales NR, Lu S, Chitsaz F, Geer LY, Geer RC, He J, Gwadz M, Hurwitz DI, Lanczycki CJ, Lu F, Marchler GH, Song JS, Thanki N, Wang Z, Yamashita RA, Zhang D, Zheng C, Bryant SH (2015). CDD: NCBI’s conserved domain database. Nucleic Acids Research.

[ref-54] Marshansky V, Rubinstein JL, Grüber G (2014). Eukaryotic V-ATPase: novel structural findings and functional insights. Biochimica et Biophysica Acta–Bioenergetics.

[ref-55] McCormack RB (2014). New records and review of the translocation of the yabby Cherax destructor into eastern drainages of New South Wales, Australia. Australian Zoologist.

[ref-56] McGrath LL, Vollmer SV, Kaluziak ST, Ayers J (2016). De novo transcriptome assembly for the lobster Homarus americanus and characterization of differential gene expression across nervous system tissues. BMC Genomics.

[ref-57] McNamara JC, Faria SC (2012). Evolution of osmoregulatory patterns and gill ion transport mechanisms in the decapod Crustacea: a review. Journal of Comparative Physiology. B, Biochemical, Systemic, and Environmental Physiology.

[ref-58] Merz KM (1990). Insights into the function of the zinc hydroxide-Thr199-Glu106 hydrogen bonding network in carbonic anhydrases. Journal of Molecular Biology.

[ref-59] Mitsunaga-Nakatsubo K, Yamazaki K, Hatoh-Okazaki M, Kawashita H, Okamura C, Akasaka K, Shimada H, Yasumasu I (1996). cDNA cloning of Na^+^, K^+^-ATPase *α*-subunit from embryos of the sea urchin, Hemicentrotus pulcherrimus. Zoological Science.

[ref-60] Muench SP, Trinick J, Harrison MA (2011). Structural divergence of the rotary ATPases. Quarterly Reviews of Biophysics.

[ref-61] Muse SV, Gaut BS (1994). A likelihood approach for comparing synonymous and nonsynonymous nucleotide substitution rates, with application to the chloroplast genome. Molecular Biology and Evolution.

[ref-62] Nei M, Kumar S (2000). Molecular evolution and phylogenetics.

[ref-63] Onken H, Graszynski K, Zeiske W (1991). Na^+^ -independent, electrogenic Cl-uptake across the posterior gills of the Chinese crab (Eriocheir sinensis): voltage-clamp and microelectrode studies. Journal of Comparative Physiology B.

[ref-64] Onken H, Tresguerres M, Luquet CM (2003). Active NaCl absorption across posterior gills of hyperosmoregulating Chasmagnathus granulatus. Journal of Experimental Biology.

[ref-65] Pan L-Q, Zhang L-J, Liu H-Y (2007). Effects of salinity and pH on ion-transport enzyme activities, survival and growth of *Litopenaeus vannamei* postlarvae. Aquaculture.

[ref-66] Petersen TN, Brunak S, Von Heijne G, Nielsen H (2011). SignalP 4.0: discriminating signal peptides from transmembrane regions. Nature Methods.

[ref-67] Pongsomboon S, Udomlertpreecha S, Amparyup P, Wuthisuthimethavee S, Tassanakajon A (2009). Gene expression and activity of carbonic anhydrase in salinity stressed *Penaeus monodon*. Comparative Biochemistry and Physiology a-Molecular & Integrative Physiology.

[ref-68] Prentis PJ, Pavasovic A (2014). The Anadara trapezia transcriptome: A resource for molluscan physiological genomics. Marine Genomics 18, Part B.

[ref-69] Rawson S, Phillips C, Huss M, Tiburcy F, Wieczorek H, Trinick J, Harrison MA, Muench SP (2015). Structure of the Vacuolar H^+^-ATPase rotary motor reveals new mechanistic insights. Structure.

[ref-70] Ren Q, Pan L, Zhao Q, Si L (2015). Ammonia and urea excretion in the swimming crab Portunus trituberculatus exposed to elevated ambient ammonia-N. Comparative Biochemistry and Physiology Part A: Molecular & Integrative Physiology.

[ref-71] Romano N, Zeng C (2012). Osmoregulation in decapod crustaceans: implications to aquaculture productivity, methods for potential improvement and interactions with elevated ammonia exposure. Aquaculture.

[ref-72] Schultz J, Milpetz F, Bork P, Ponting CP (1998). SMART, a simple modular architecture research tool: ddentification of signaling domains. Proceedings of the National Academy of Sciences of the United States of America.

[ref-73] Sergei LKP, Muse SV (2005). HyPhy: hypothesis testing using phylogenies, Statistical methods in molecular evolution.

[ref-74] Serrano L, Halanych KM, Henry RP (2007). Salinity-stimulated changes in expression and activity of two carbonic anhydrase isoforms in the blue crab *Callinectes sapidus*. Journal of Experimental Biology.

[ref-75] Serrano L, Henry RP (2008). Differential expression and induction of two carbonic anhydrase isoforms in the gills of the euryhaline green crab, *Carcinus maenas*, in response to low salinity. Comparative Biochemistry and Physiology Part D: Genomics and Proteomics.

[ref-76] Shen H, Hu Y, Ma Y, Zhou X, Xu Z, Shui Y, Li C, Xu P, Sun X (2014). In-depth transcriptome analysis of the red swamp crayfish Procambarus clarkii. PLOS ONE.

[ref-77] Shrivastava AN, Redeker V, Fritz N, Pieri L, Almeida LG, Spolidoro M, Liebmann T, Bousset L, Renner M, Léna C, Aperia A (2015). *α*-synuclein assemblies sequester neuronal *α*3-Na^+^/K^+^-ATPase and impair Na^+^ gradient. The EMBO Journal.

[ref-78] Sigrist CJA, De Castro E, Cerutti L, Cuche BA, Hulo N, Bridge A, Bougueleret L, Xenarios I (2013). New and continuing developments at PROSITE. Nucleic Acids Research.

[ref-79] Suzuki Y, Gojobori T (1999). A method for detecting positive selection at single amino acid sites. Molecular Biology and Evolution.

[ref-80] Tajima F (1993). Simple methods for testing the molecular evolutionary clock hypothesis. Genetics.

[ref-81] Tamura K, Stecher G, Peterson D, Filipski A, Kumar S (2013). MEGA6: molecular evolutionary genetics analysis version 6.0. Molecular Biology and Evolution.

[ref-82] Tan MH, Gan HM, Gan HY, Lee YP, Croft LJ, Schultz MB, Miller AD, Austin CM (2016). First comprehensive multi-tissue transcriptome of Cherax quadricarinatus (Decapoda: Parastacidae) reveals unexpected diversity of endogenous cellulase. Organisms Diversity & Evolution.

[ref-83] Towle DW, Henry RP, Terwilliger NB (2011). Microarray-detected changes in gene expression in gills of green crabs (*Carcinus maenas*) upon dilution of environmental salinity. Comparative Biochemistry and Physiology Part D: Genomics and Proteomics.

[ref-84] Towle DW, Weihrauch D (2001). Osmoregulation by gills of euryhaline crabs: molecular analysis of transporters. American Zoologist.

[ref-85] Towle DW, Weihrauch D, Fooks A, Cohen ZS (1997). Molecular identification and cDNA sequencing of a Na^+^/K^+^/2Cl–cotransporter in gills of the euryhaline blue crab Callinectes sapidus. American Zoologist.

[ref-86] Uda K, Fujimoto N, Akiyama Y, Mizuta K, Tanaka K, Ellington WR, Suzuki T (2006). Evolution of the arginine kinase gene family. Comparative Biochemistry and Physiology Part D: Genomics and Proteomics.

[ref-87] Visudtiphole V, Watthanasurorot A, Klinbunga S, Menasveta P, Kirtikara K (2010). Molecular characterization of Calreticulin: A biomarker for temperature stress responses of the giant tiger shrimp Penaeus monodon. Aquaculture.

[ref-88] Wang L, Wang W-N, Liu Y, Cai D-X, Li J-Z, Wang A-L (2012). Two types of ATPases from the Pacific white shrimp, Litopenaeus vannamei in response to environmental stress. Molecular Biology Reports.

[ref-89] Watthanasurorot A, Jiravanichpaisal P, Söderhäll K, Söderhäll I (2013). A calreticulin/gC1qR complex prevents cells from dying: a conserved mechanism from arthropods to humans. Journal of Molecular Cell Biology.

[ref-90] Weihrauch D, McNamara JC, Towle DW, Onken H (2004). Ion-motive ATPases and active, transbranchial NaCl uptake in the red freshwater crab, *Dilocarcinus pagei* (Decapoda, Trichodactylidae). Journal of Experimental Biology.

[ref-91] Wu H, Che X, Tang J, Ma F, Pan K, Zhao M, Shao A, Wu Q, Zhang J, Hong Y (2015). The K^+^-Cl− Cotransporter KCC2 and Chloride homeostasis: potential therapeutic target in acute central nervous system injury. Molecular Neurobiology.

[ref-92] Xie Z, Askari A (2002). Na^+^/K^+^-ATPase as a signal transducer. European Journal of Biochemistry.

[ref-93] Xu Q, Liu Y (2011). Gene expression profiles of the swimming crab Portunus trituberculatus exposed to salinity stress. Marine Biology.

[ref-94] Xie Y, Gong J, Ye H, Huang H, Yang YN (2014). Molecular characteristic and responsive expression of arginine kinase in the mud crab, scylla paramamosain. Journal of the World Aquaculture Society.

[ref-95] Xu B, Long C, Dong W, Shao Q, Shu M (2015). Molecular characterization of calreticulin gene in mud crab Scylla paramamosain (Estampador): implications for the regulation of calcium homeostasis during moult cycle. Aquaculture Research.

[ref-96] Yuan Z, Cai T, Tian J, Ivanov AV, Giovannucci DR, Xie Z (2005). Na/K-ATPase tethers phospholipase C and IP3 receptor into a calcium-regulatory complex. Molecular Biology of the Cell.

[ref-97] Zhang M, Gaschen B, Blay W, Foley B, Haigwood N, Kuiken C, Korber B (2004). Tracking global patterns of N-linked glycosylation site variation in highly variable viral glycoproteins: HIV, SIV, and HCV envelopes and influenza hemagglutinin. Glycobiology.

[ref-98] Zhang X, Li Y, Zhang X, Duan Z, Zhu J (2015). Regulation of transepithelial ion transport in the rat late distal colon by the sympathetic nervous system. Physiological Research/Academia Scientiarum Bohemoslovaca.

[ref-99] Zuckerkandl E, Pauling L, Bryson V, Vogel HJ (1965). Evolutionary divergence and convergence in proteins. Evolving Genes and Proteins.

