# Supplementary material for: Comparative molecular analyses of select pH- and osmoregulatory genes in three freshwater crayfish Cherax quadricarinatus, C. destructor and C. cainii
Source: PeerJ. 2017 Aug 24;5:e3623. doi: 10.7717/peerj.3623 (PMC5572425; doi:10.7717/peerj.3623)
Supplement: Table S1 — Identity matrix based on amino acid composition between three Cherax species (Cherax quadricarnatus, Cherax cainii and Cherax destructor) and other crustaceans. The first row and first column indicate species. Values in the cells indicate identity in percentage. CAc, Cytoplasmic carbonic anhydrase; CAg, GPI-linked carbonic anhydrase; CAb, Beta carbonic anhydrase; NKA, Na+/K+-ATPase alpha subunit; HAT, Vacuolar type H+-ATPase 116 kda; NKCC, Na+/K+/2Cl− cotransporter; NBC, Na+/HCO${}_{3}^{-}$3− cotransporter; NHE, Na+/H+ exchanger 3; NCX, Na+/Ca+2 exchanger 1; AK, Arginine kinase; and CRT, Calreticulin. [file peerj-05-3623-s001.docx]

## Supplementary Table 1: Identity matrix across species based on amino acid compositions (S Table 1.1-1.11)

S Table 1.1. Amino acid identity across crustacean species based on cytoplasmic CA (CAc)

|  | *C. quadricarinatus* | *C. cainii* | *C. destructor* | *L. vannamei* | *P. monodon* | *C. sapidus* | *P. trituberculatus* |
| --- | --- | --- | --- | --- | --- | --- | --- |
| *Cherax quadricarinatus CAc (KM538165)* |  | 97.786 | 97.786 | 76.015 | 76.015 | 73.801 | 73.063 |
| *Cherax cainii CAc (KP221715)* | 97.786 |  | 97.786 | 76.015 | 76.015 | 73.801 | 73.063 |
| *Cherax destructor (KP299962)* | 97.786 | 97.786 |  | 75.646 | 75.277 | 73.801 | 72.694 |
| *Litopenaeus vannamei (HM991703)* | 76.015 | 76.015 | 75.646 |  | 97.778 | 73.432 | 73.063 |
| *Penaeus monodon (EF672697)* | 76.015 | 76.015 | 75.277 | 97.778 |  | 73.432 | 72.694 |
| *Callinectes sapidus (EF375490)* | 73.801 | 73.801 | 73.801 | 73.432 | 73.432 |  | 94.465 |
| *Portunus trituberculatus (JX524149)* | 73.063 | 73.063 | 72.694 | 73.063 | 72.694 | 94.465 |  |

S Table 1.2. Amino acid identity across crustacean species based on membrane-associated CA (CAg)

|  | *C. quadricarinatus* | *C. cainii* | *C. destructor* | *L. vannamei* | *H. rubra* | *C. sapidus* | *P. trituberculatus* | *C. maenas* |
| --- | --- | --- | --- | --- | --- | --- | --- | --- |
| *Cherax quadricarinatus (KM538166)* |  | 99.355 | 92.929 | 71.104 | 72.903 | 73.701 | 73.377 | 70.13 |
| *Cherax cainii (KP221716)* | 99.355 |  | 92.256 | 71.429 | 73.226 | 73.701 | 73.377 | 70.455 |
| *Cherax destructor (KP299963)* | 92.929 | 92.256 |  | 66.78 | 68.35 | 68.475 | 68.136 | 67.119 |
| *Litopenaeus vannamei (JX975725)* | 71.104 | 71.429 | 66.78 |  | 75.806 | 70.779 | 69.481 | 69.481 |
| *Halocaridina rubra (KF650061)* | 72.903 | 73.226 | 68.35 | 75.806 |  | 71.935 | 71.613 | 70.645 |
| *Callinectes sapidus (EF375491)* | 73.701 | 73.701 | 68.475 | 70.779 | 71.935 |  | 95.13 | 88.636 |
| *Portunus trituberculatus (JX524150)* | 73.377 | 73.377 | 68.136 | 69.481 | 71.613 | 95.13 |  | 87.338 |
| *Carcinus maenas (EU273944)* | 70.13 | 70.455 | 67.119 | 69.481 | 70.645 | 88.636 | 87.338 |  |

S Table 1.3. Amino acid identity across crustacean species based on beta CA (CA-beta)

|  | *C. quadricarinatus* | *C. cainii* | *C. destructor* | *B. terrestris* | *Apis dorsata* | *N. vitripennis* | *Athalia rosae* |
| --- | --- | --- | --- | --- | --- | --- | --- |
| *Cherax quadricarinatus (KM538167)* |  | 99.222 | 98.833 | 62.745 | 60.392 | 63.529 | 63.137 |
| *Cherax cainii (KP221717)* | 99.222 |  | 98.054 | 63.137 | 60.784 | 63.922 | 63.529 |
| *Cherax destructor (KP299965)* | 98.833 | 98.054 |  | 63.137 | 61.176 | 64.314 | 63.922 |
| *Bombus terrestris (XM_003402502)* | 62.745 | 63.137 | 63.137 |  | 82.745 | 84.706 | 86.275 |
| *Apis dorsata (XM_006612942)* | 60.392 | 60.784 | 61.176 | 82.745 |  | 81.569 | 83.529 |
| *Nasonia vitripennis (XM_001606922)* | 63.529 | 63.922 | 64.314 | 84.706 | 81.569 |  | 94.902 |
| *Athalia rosae (XM 012404250)* | 63.137 | 63.529 | 63.922 | 86.275 | 83.529 | 94.902 |  |

S Table 1.4. Amino acid identity across crustacean species based on Na^+^/K^+^-ATPase alpha subunit (NKA)

|  | *C. quadricarnatus NKA* | *Cherax cainii (KP221718)* | *C. destructor* | *P. monodon* | *F. indicus* | *L. stylirostris* | *C. sapidus* | *P. trituberculatus* | *P. marmoratus* | *E. sinensis* |
| --- | --- | --- | --- | --- | --- | --- | --- | --- | --- | --- |
| *Cherax quadricarnatus NKA* |  | 99.904 | 99.711 | 93.545 | 94.027 | 91.908 | 95 | 95 | 95.568 | 95.665 |
| *Cherax cainii (KP221718)* | 99.904 |  | 99.807 | 93.545 | 94.027 | 92.004 | 95.096 | 95.096 | 95.665 | 95.761 |
| *Cherax destructor (KP299966)* | 99.711 | 99.807 |  | 93.449 | 93.931 | 91.908 | 95 | 95 | 95.568 | 95.665 |
| *Penaeus monodon (\|DQ399797)* | 93.545 | 93.545 | 93.449 |  | 98.844 | 95.472 | 92.212 | 92.404 | 92.1 | 92.004 |
| *Fenneropenaeus indicus (HM012803)* | 94.027 | 94.027 | 93.931 | 98.844 |  | 96.243 | 92.596 | 92.788 | 92.775 | 92.293 |
| *Litopenaeus stylirostris (JN561324)* | 91.908 | 92.004 | 91.908 | 95.472 | 96.243 |  | 90.577 | 90.865 | 90.655 | 90.366 |
| *Callinectes sapidus (AF327439)* | 95 | 95.096 | 95 | 92.212 | 92.596 | 90.577 |  | 99.326 | 97.69 | 97.209 |
| *Portunus trituberculatus (JX173959)* | 95 | 95.096 | 95 | 92.404 | 92.788 | 90.865 | 99.326 |  | 97.594 | 97.113 |
| *Pachygrapsus marmoratus (DQ173924)* | 95.568 | 95.665 | 95.568 | 92.1 | 92.775 | 90.655 | 97.69 | 97.594 |  | 98.457 |
| *Eriocheir sinensis (KC691291)* | 95.665 | 95.761 | 95.665 | 92.004 | 92.293 | 90.366 | 97.209 | 97.113 | 98.457 |  |

S Table 1.5. Amino acid identity across crustacean species based on V- type H^+^-ATPase (HAT-A)

|  | *C. quadricarinatus* | *C. destructor* | *C. cainii* | *W. auropunctata* | *P. barbatus* | *C. floridanus* | *B. terrestris* |
| --- | --- | --- | --- | --- | --- | --- | --- |
| *Cherax quadricarinatus* |  | 99.158 | 98.676 | 71.059 | 70.824 | 71.025 | 71.226 |
| *Cherax.destructor (KP299969)* | 99.158 |  | 99.278 | 70.824 | 70.588 | 70.907 | 70.991 |
| *Cherax.cainii (KP221721)* | 98.676 | 99.278 |  | 70.824 | 70.588 | 70.907 | 70.991 |
| *Wasmannia auropunctata (XM 011701129)* | 71.059 | 70.824 | 70.824 |  | 96.908 | 95.838 | 88.889 |
| *Pogonomyrmex barbatus (XM 011643683)* | 70.824 | 70.588 | 70.588 | 96.908 |  | 95.244 | 88.416 |
| *Camponotus floridanus (XM 011263300)* | 71.025 | 70.907 | 70.907 | 95.838 | 95.244 |  | 89.125 |
| *Bombus terrestris (XM 003397650)* | 71.226 | 70.991 | 70.991 | 88.889 | 88.416 | 89.125 |  |

S Table 1.6. Amino acid identity across crustacean species based on Na^+^/K^+^/2Cl^-^ cotransporter (NKCC)

|  | *C. quadricarinatus* | *C. destructor* | *C. cainii* | *C. sapidus* | *H. rubra* | *M. domestica* | *B. cucurbitae* | *D. melanogaster* | *B. terrestris* |
| --- | --- | --- | --- | --- | --- | --- | --- | --- | --- |
| *Cherax quadricarinatus* |  | 96.934 | 97.288 | 49.25 | 49.024 | 49.664 | 49.943 | 50 | 47.119 |
| *Cherax destructor (KP299986)* | 96.934 |  | 97.03 | 50.431 | 49.893 | 51.207 | 51.386 | 51.86 | 48.837 |
| *Cherax cainii (KP221733)* | 97.288 | 97.03 |  | 48.448 | 47.711 | 48.802 | 49.101 | 49.282 | 47.25 |
| *Callinectes sapidus (AF190129)* | 49.25 | 50.431 | 48.448 |  | 76.245 | 49.359 | 49.118 | 49.722 | 47.903 |
| *Halocaridinarubra (KF650065)* | 49.024 | 49.893 | 47.711 | 76.245 |  | 48.168 | 48.37 | 48.597 | 47.23 |
| *Musca domestica (XM_011298324)* | 49.664 | 51.207 | 48.802 | 49.359 | 48.168 |  | 84.898 | 81.795 | 57.713 |
| *Bactrocera cucurbitae (XM 011181369)* | 49.943 | 51.386 | 49.101 | 49.118 | 48.37 | 84.898 |  | 81.803 | 58.079 |
| *Drosophila melanogaster (NM_140315)* | 50 | 51.86 | 49.282 | 49.722 | 48.597 | 81.795 | 81.803 |  | 58.318 |
| *Bombus terrestris (XM 003399442)* | 47.119 | 48.837 | 47.25 | 47.903 | 47.23 | 57.713 | 58.079 | 58.318 |  |

S Table 1.7. Amino acid identity across crustacean species based on Na^+^/HCO_3_^-^ cotransporter (NBC)

|  | *C. destructor* | *C. destructor* | *C. cainii* | *C. cainii* | *C. quadricarinatus* | *W. auropunctata* | *P. barbatus* |
| --- | --- | --- | --- | --- | --- | --- | --- |
| *Cherax destructor (KP299976)* |  | 99.914 | 98.497 | 98.361 | 97.412 | 60.371 | 59.354 |
| *Cherax destructor (KP299977)* | 99.914 |  | 98.361 | 98.447 | 97.498 | 60.371 | 59.354 |
| *Cherax cainii (KP221727)* | 98.497 | 98.361 |  | 99.914 | 97.323 | 60.455 | 59.437 |
| *Cherax cainii (KP221728)* | 98.361 | 98.447 | 99.914 |  | 97.409 | 60.455 | 59.437 |
| *Cherax quadricarinatus (KP221728)* | 97.412 | 97.498 | 97.323 | 97.409 |  | 60.796 | 59.933 |
| *Wasmannia auropunctata (XM 011705063)* | 60.371 | 60.371 | 60.455 | 60.455 | 60.796 |  | 94.187 |
| *Pogonomyrmex barbatus (XM 011642543)* | 59.354 | 59.354 | 59.437 | 59.437 | 59.933 | 94.187 |  |

S Table 1.8. Amino acid identity across crustacean species based on Na^+^/H^+^ exchanger (NHE)

|  | *C. quadricarinatus* | *C. destructor* | *C. cainii* | *A. echinatior* | *A. echinatior* | *V. emeryi* | *P. barbatus* | *B. impatiens* |
| --- | --- | --- | --- | --- | --- | --- | --- | --- |
| *Cherax quadricarinatus (KM880153)* |  | 92.628 | 91.161 | 40.833 | 40.535 | 40.457 | 40.99 | 40.04 |
| *Cherax destructor (KP299982)* | 92.628 |  | 92.259 | 41.071 | 40.774 | 40.597 | 41.089 | 40.681 |
| *Cherax cainii (KP221730)* | 91.161 | 92.259 |  | 39.603 | 39.319 | 39.242 | 39.754 | 38.725 |
| *Acromyrmex echinatior (XM_011069477)* | 40.833 | 41.071 | 39.603 |  | 96.753 | 91.516 | 92.769 | 78.524 |
| *Acromyrmex echinatior (XM_011069496)* | 40.535 | 40.774 | 39.319 | 96.753 |  | 88.37 | 89.534 | 76.42 |
| *Vollenhovia emeryi (XM 012027471)* | 40.457 | 40.597 | 39.242 | 91.516 | 88.37 |  | 90.095 | 78.178 |
| *Pogonomyrmex barbatus (XM_011649419)* | 40.99 | 41.089 | 39.754 | 92.769 | 89.534 | 90.095 |  | 79.037 |
| *Bombus impatiens (XM 003491441)* | 40.04 | 40.681 | 38.725 | 78.524 | 76.42 | 78.178 | 79.037 |  |

S Table 1.9. Amino acid identity across crustacean species based on Na^+^/Ca^+2^ exchanger (NCX)

|  | *C. destructor* | *C. cainii* | *C. quadricarinatus* | *P. barbatus* | *A. cephalotes* | *W. auropunctata* | *C. floridanus* | *A. dorsata* |
| --- | --- | --- | --- | --- | --- | --- | --- | --- |
| *Cherax destructor (KP299983)* |  | 98.94 | 98.469 | 38.317 | 37.721 | 38 | 37.751 | 37.458 |
| *Cherax cainii (KP221731)* | 98.94 |  | 98.231 | 38.206 | 37.611 | 37.889 | 37.639 | 37.347 |
| *Cherax quadricarinatus* | 98.469 | 98.231 |  | 38.206 | 37.611 | 37.889 | 37.639 | 37.458 |
| *Pogonomyrmex barbatus (XM 011636160)* | 38.317 | 38.206 | 38.206 |  | 95.293 | 93.8 | 91.734 | 87.011 |
| *Atta cephalotes (XM 012203310)* | 37.721 | 37.611 | 37.611 | 95.293 |  | 95.293 | 93.456 | 87.371 |
| *Wasmannia auropunctata (XM 011701097)* | 38 | 37.889 | 37.889 | 93.8 | 95.293 |  | 93.318 | 87.112 |
| *Camponotus floridanus (XM_011255402)* | 37.751 | 37.639 | 37.639 | 91.734 | 93.456 | 93.318 |  | 87.112 |
| *Apis dorsata (XM 006616508)* | 37.458 | 37.347 | 37.458 | 87.011 | 87.371 | 87.112 | 87.112 |  |

S Table 1.10. Amino acid identity across crustacean species based on Arginine kinase

|  | *C. quadricarinatus* | *C. destructor* | *C. cainii* | *P. clarkii* | *H. vulgaris* | *C. maenas* | *S. serrata* | *C. sapidus* | *L. vannamei* | *P. monodon* |
| --- | --- | --- | --- | --- | --- | --- | --- | --- | --- | --- |
| *Cherax.quadricarinatus* |  | 98.599 | 98.039 | 96.639 | 94.944 | 92.717 | 92.135 | 92.997 | 90.169 | 90.73 |
| *Cherax destructor (KP299970)* | 98.599 |  | 97.479 | 96.359 | 94.663 | 92.437 | 92.135 | 92.717 | 89.607 | 89.607 |
| *Cherax cainii (KP221722)* | 98.039 | 97.479 |  | 96.078 | 94.101 | 92.157 | 91.573 | 91.877 | 89.045 | 89.607 |
| *Procambarus clarkii (JN828651)* | 96.639 | 96.359 | 96.078 |  | 95.225 | 93.838 | 94.382 | 94.678 | 91.292 | 91.011 |
| *Homarus vulgaris (X68703)* | 94.944 | 94.663 | 94.101 | 95.225 |  | 92.697 | 92.697 | 93.82 | 91.011 | 91.011 |
| *Carcinus maenas (AF167313)* | 92.717 | 92.437 | 92.157 | 93.838 | 92.697 |  | 97.191 | 97.199 | 91.292 | 92.135 |
| *Scylla serrata (GQ851626)* | 92.135 | 92.135 | 91.573 | 94.382 | 92.697 | 97.191 |  | 98.034 | 93.258 | 94.101 |
| *Callinectes sapidus (AF233355)* | 92.997 | 92.717 | 91.877 | 94.678 | 93.82 | 97.199 | 98.034 |  | 92.978 | 92.978 |
| *Litopenaeus vannamei (DQ975203)* | 90.169 | 89.607 | 89.045 | 91.292 | 91.011 | 91.292 | 93.258 | 92.978 |  | 96.067 |
| *Penaeus monodon (KF177337)* | 90.73 | 89.607 | 89.607 | 91.011 | 91.011 | 92.135 | 94.101 | 92.978 | 96.067 |  |

S Table 1.11. Amino acid identity across crustacean species based on Calreticulin

|  | *C. quadricarinatus* | *C. destructor* | *C. cainii* | *F. chinensis* | *P. monodon* | *L. vannamei* | *E. carinicauda* | *P. leniusculus* | *S. paramamosain* |
| --- | --- | --- | --- | --- | --- | --- | --- | --- | --- |
| *Cherax quadricarinatus (KM538170)* |  | 98.759 | 99.005 | 86.946 | 87.192 | 86.453 | 85.714 | 91.832 | 86.207 |
| *Cherax destructor (KP299971)* | 98.759 |  | 98.263 | 86.7 | 86.946 | 86.207 | 85.961 | 91.111 | 85.714 |
| *Cherax cainii (KP221723)* | 99.005 | 98.263 |  | 87.192 | 87.192 | 86.453 | 85.961 | 91.337 | 86.207 |
| *Fenneropenaeus chinensis (DQ323054)* | 86.946 | 86.7 | 87.192 |  | 98.768 | 96.798 | 88.424 | 86.7 | 85.222 |
| *Penaeus monodon (GU140040)* | 87.192 | 86.946 | 87.192 | 98.768 |  | 98.03 | 88.67 | 86.946 | 83.99 |
| *Litopenaeus vannamei (JQ682618.)* | 86.453 | 86.207 | 86.453 | 96.798 | 98.03 |  | 88.177 | 86.207 | 83.99 |
| *Exopalaemon carinicauda (JX508647)* | 85.714 | 85.961 | 85.961 | 88.424 | 88.67 | 88.177 |  | 85.222 | 83.99 |
| *Pacifastacus leniusculus (HQ596362)* | 91.832 | 91.111 | 91.337 | 86.7 | 86.946 | 86.207 | 85.222 |  | 84.975 |
| *Scylla paramamosain (HQ260918)* | 86.207 | 85.714 | 86.207 | 85.222 | 83.99 | 83.99 | 83.99 | 84.975 |  |
