# Supplementary material for: Comparative molecular analyses of select pH- and osmoregulatory genes in three freshwater crayfish Cherax quadricarinatus, C. destructor and C. cainii
Source: PeerJ. 2017 Aug 24;5:e3623. doi: 10.7717/peerj.3623 (PMC5572425; doi:10.7717/peerj.3623)
Supplement: Table S2 — Information on whether the genes are present or not in particular tissue. ‘p’ in the celll indicates present and ‘a’ indicates absent. A total of eighty (80) most important genes associated with pH balance and osmo-regulation were observed. [file peerj-05-3623-s002.docx]

## Supplementary Table 2: Tissue-specific expression of major pH- and osmoregulatory genes in Redclaw crayfish *Cherax quadricarinatus*

| **#** | **Gene Name** | **Gill** | **Hepatopancreas** | **Heart** | **Kidney** | **Liver** | **Nerve** | **Testes** |
| --- | --- | --- | --- | --- | --- | --- | --- | --- |
| 1 | cytoplasmic carbonic anhydrase (CAc) | p | p | p | p | p | p | p |
| 2 | GPI-linked carbonic anhydrase (CAg) | p | p | p | p | p | p | p |
| 3 | Beta carbonic anhydrase (CAb) | p | a | p | p | p | p | a |
| 4 | carbonic anhydrase-related protein 10 | p | a | a | a | a | p | a |
| 5 | na+ k+-atpase alpha subunit | p | p | p | p | p | p | p |
| 6 | sodium potassium-transporting atpase subunit beta | p | p | p | p | p | p | p |
| 7 | v-type proton atpase catalytic subunit a | p | p | p | p | p | p | p |
| 8 | v-type proton atpase 116 kda subunit a | p | p | p | a | p | p | p |
| 9 | v-type proton atpase subunit B | p | p | p | a | a | a | a |
| 10 | v-type proton atpase subunit C | p | p | p | a | p | a | a |
| 11 | v-type proton atpase subunit D | p | p | p | p | p | p | p |
| 12 | v-type proton atpase subunit E | p | p | p | p | p | p | p |
| 13 | v-type proton atpase subunit H | p | p | a | a | a | a | a |
| 14 | v-type proton atpase subunit S | p | p | p | p | p | p | a |
| 15 | v-type proton atpase 21 kda proteolipid subunit | p | p | p | p | p | p | p |
| 16 | arginine kinase | p | p | p | p | p | p | p |
| 17 | alkaline phosphatase | p | p | p | p | p | p | p |
| 18 | calreticulin | p | p | p | p | p | p | p |
| 19 | selenophosphate synthetase | p | p | p | p | p | p | p |
| 20 | calcium-transporting atpase sarcoplasmic endoplasmic reticulum type | p | p | p | p | p | p | p |
| 21 | calcium-transporting atpase type 2C | p | p | p | p | p | p | p |
| 22 | plasma membrane calcium-transporting atpase 3 | p | p | p | p | p | p | p |
| 23 | sodium hydrogen exchanger 2 | a | a | a | a | a | a | p |
| 24 | sodium hydrogen exchanger 3 | p | a | a | p | a | p | a |
| 25 | sodium hydrogen exchanger 7 | p | p | p | p | p | p | p |
| 26 | sodium hydrogen exchanger 8 | p | p | p | p | p | p | p |
| 27 | sodium hydrogen exchanger 9 | p | a | a | a | a | p | a |
| 28 | sodium calcium exchanger 1 | p | a | p | p | p | p | p |
| 29 | sodium calcium exchanger 3 | a | a | p | p | p | p | a |
| 30 | sodium bicarbonate cotransporter (Na+/HCO3) | p | a | p | p | p | p | p |
| 31 | sodium-driven chloride bicarbonate exchanger (Cl (Na+)/HCO3) | p | p | p | a | p | a | a |
| 32 | sodium chloride cotransporter (Na/Cl) | p | p | p | p | p | p | p |
| 33 | potassium chloride symporter (K/Cl) | p | p | p | p | a | p | a |
| 34 | sodium channel protein (Na) | a | a | p | a | a | p | a |
| 35 | sodium channel protein 60e | a | a | a | a | a | p | p |
| 36 | potassium channels protein 7 (t family ) | a | a | a | a | p | p | a |
| 37 | potassium channels protein 18 (t family ) | p | a | a | a | p | p | p |
| 38 | potassium channel 1 (k family) | a | p | a | a | p | p | a |
| 39 | potassium channel 3 (k family) | a | a | a | a | a | p | a |
| 40 | potassium channel 9 (k family) | a | a | a | a | a | p | p |
| 41 | potassium channel 10 (k family) | a | a | a | a | a | p | a |
| 42 | potassium channel 16 (k family) | p | a | p | a | p | a | a |
| 43 | potassium channel 18 (k family) | a | a | a | a | a | a | p |
| 44 | calcium-activated potassium channel | a | a | p | p | a | p | p |
| 45 | two pore potassium channel protein sup-9 | a | a | a | P | p | p | p |
| 46 | chloride channel | p | p | p | p | a | p | p |
| 47 | chloride channel protein 2 | p | p | p | p | p | p | p |
| 48 | chloride channel protein 3 | p | a | a | p | p | p | p |
| 49 | chloride channel protein 7 | a | a | a | p | a | a | a |
| 50 | epithelial chloride channel | p | p | p | p | p | p | p |
| 51 | glutamate-gated chloride channel | p | p | a | a | a | p | a |
| 52 | histamine-gated chloride channel | a | a | p | a | p | a | a |
| 53 | ph-sensitive chloride channel | a | a | a | p | p | a | a |
| 54 | ca-activated cl channel protein | p | p | p | p | p | p | p |
| 55 | two pore calcium channel protein 1 | p | p | p | p | a | p | p |
| 56 | calcium release-activated calcium channel protein | p | p | p | p | p | p | p |
| 57 | Calmodulin | p | p | p | p | p | p | p |
| 58 | h+ transporting atp synthase beta subunit | p | p | a | p | a | p | p |
| 59 | h+ transporting atp synthase subunit o | p | p | p | p | p | p | p |
| 60 | h+ transporting atp synthase subunit d | p | p | p | p | p | p | p |
| 61 | Integrin alpha | p | p | p | p | p | p | p |
| 62 | Integrin beta | p | p | p | p | p | p | p |
| 63 | ATP-binding cassette transporters A | p | p | p | p | p | p | p |
| 64 | ATP-binding cassette transporters B | p | p | p | p | p | p | p |
| 65 | ATP-binding cassette transporters C | p | p | p | p | p | p | a |
| 66 | ATP-binding cassette transporters D | p | p | p | p | p | p | p |
| 67 | ATP-binding cassette transporters E | p | p | p | p | p | p | p |
| 68 | ATP-binding cassette transporters F | p | p | p | p | p | p | p |
| 69 | ATP-binding cassette transporters G | p | p | p | p | a | p | p |
| 70 | Calmodulin | p | p | p | p | p | p | p |
| 71 | p38 mitogen-activated protein kinase | p | p | p | p | p | p | p |
| 72 | Aquaporin transporter | p | p | p | p | p | p | p |
| 73 | Aquaporin-1 | a | a | p | a | a | a | a |
| 74 | Aquaporin-3 | a | P | a | p | a | p | a |
| 75 | Aquaporin 9 | p | a | a | a | a | p | a |
| 76 | Aquaporin 10 | p | a | a | a | a | a | a |
| 77 | Aquaporin 12a | a | P | a | a | a | a | a |
| 78 | magnesium transporter protein 1 | p | p | p | p | p | p | p |
| 79 | magnesium transporter nipa | p | p | a | p | p | p | p |
| 80 | membrane magnesium transporter | p | p | p | p | p | p | p |
